# Neuroimaging findings in preclinical amyotrophic lateral sclerosis models—How well do they mimic the clinical phenotype? A systematic review

**DOI:** 10.3389/fvets.2023.1135282

**Published:** 2023-05-02

**Authors:** Amelia Elaine Cannon, Wolfgang Emanuel Zürrer, Charlotte Zejlon, Zsolt Kulcsar, Sebastian Lewandowski, Fredrik Piehl, Tobias Granberg, Benjamin Victor Ineichen

**Affiliations:** ^1^Center for Reproducible Science, University of Zurich, Zurich, Switzerland; ^2^Department of Neuroradiology, Karolinska University Hospital, Stockholm, Sweden; ^3^Department of Neuroradiology, Clinical Neuroscience Center, University Hospital Zurich, University of Zurich, Zurich, Switzerland; ^4^Department of Clinical Neuroscience, Karolinska Institutet, Stockholm, Sweden; ^5^Center of Neurology, Academic Specialist Center, Stockholm Health Services, Stockholm, Sweden

**Keywords:** motor neuron disease (MND), magnetic resonance imaging (MRI), systematic review, amyotrophic lateral sclerosis, neuroimaging, external validity, 3R, neuroscience

## Abstract

**Background and objectives:**

Animal models for motor neuron diseases (MND) such as amyotrophic lateral sclerosis (ALS) are commonly used in preclinical research. However, it is insufficiently understood how much findings from these model systems can be translated to humans. Thus, we aimed at systematically assessing the translational value of MND animal models to probe their external validity with regards to magnetic resonance imaging (MRI) features.

**Methods:**

In a comprehensive literature search in PubMed and Embase, we retrieved 201 unique publications of which 34 were deemed eligible for qualitative synthesis including risk of bias assessment.

**Results:**

ALS animal models can indeed present with human ALS neuroimaging features: Similar to the human paradigm, (regional) brain and spinal cord atrophy as well as signal changes in motor systems are commonly observed in ALS animal models. Blood-brain barrier breakdown seems to be more specific to ALS models, at least in the imaging domain. It is noteworthy that the G93A-SOD1 model, mimicking a rare clinical genotype, was the most frequently used ALS proxy.

**Conclusions:**

Our systematic review provides high-grade evidence that preclinical ALS models indeed show imaging features highly reminiscent of human ALS assigning them a high external validity in this domain. This opposes the high attrition of drugs during bench-to-bedside translation and thus raises concerns that phenotypic reproducibility does not necessarily render an animal model appropriate for drug development. These findings emphasize a careful application of these model systems for ALS therapy development thereby benefiting refinement of animal experiments.

**Systematic review registration:**

https://www.crd.york.ac.uk/PROSPERO/, identifier: CRD42022373146.

## 1. Introduction

Preclinical neuroscience has advanced our understanding of the pathophysiology of neurological diseases, and research in animal models of these diseases has identified many putative treatment targets for human diseases. However, this progress stands in stark contrast to the high attrition rates in drug development, being among the highest in neuroscience (1–4). This gap in bench-to-bedside translation can be attributed to multiple factors (5, 6), some of them inherent to the challenge of developing innovative therapies (7). However, the inappropriate design and conduct of preclinical studies have been flagged as major concerns (8–10). To this end, some attention has focused on external validity (11), i.e., the extent to which an experimental finding can be extrapolated to other settings, e.g., translation from animals to humans (12, 13).

**Figure 1:**
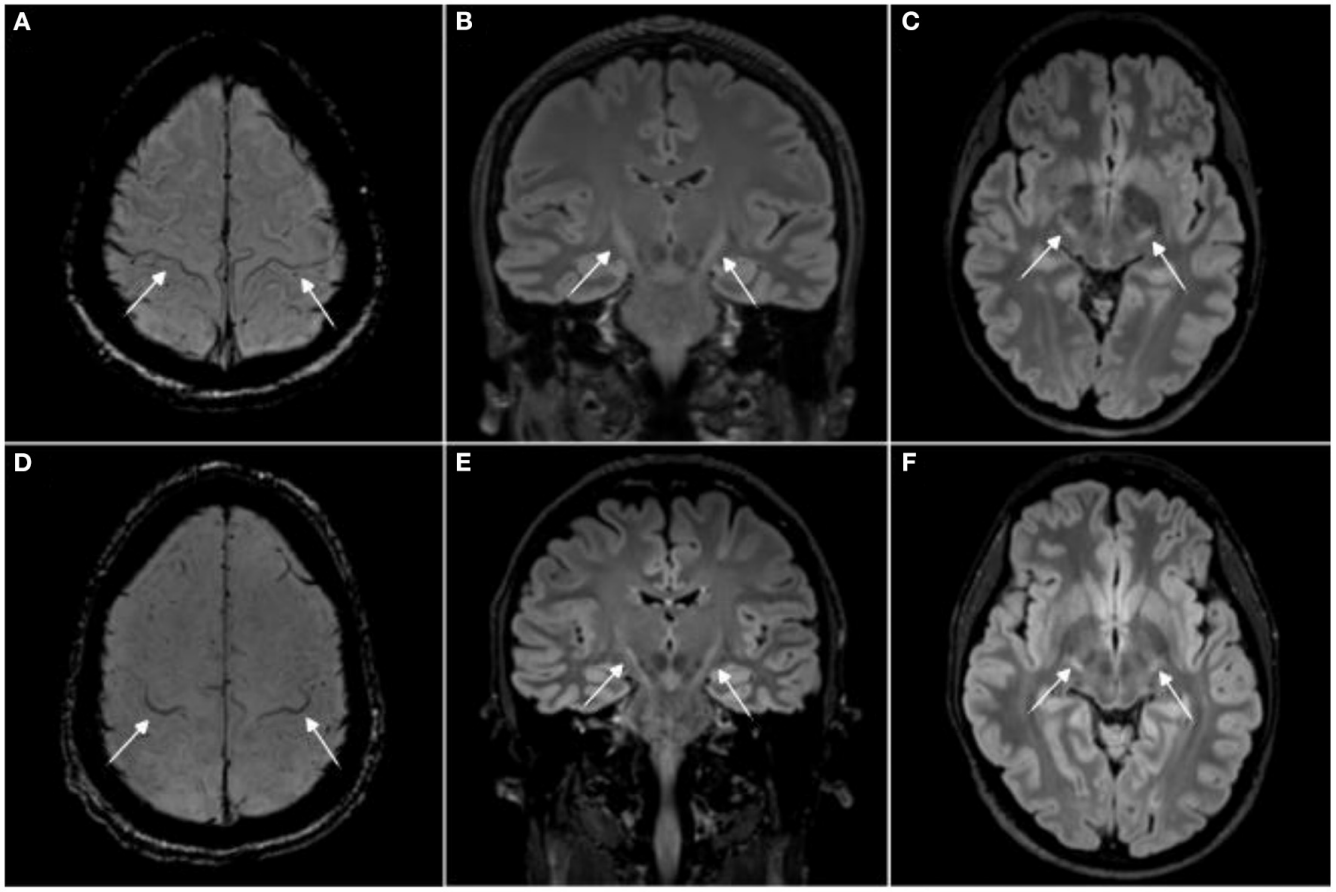
Magnetic resonance imaging signs in human amyotrophic lateral sclerosis (ALS). Magnetic resonance imaging (MRI) from two amyotrophic lateral sclerosis (ALS) patients with the “motor band sign,” i.e., motor cortex hypointensities, on susceptibility weighted imaging [SWI, **(A, D)**] and T2 hyperintensities along the corticospinal tract on 3T 3D T2w-FLAIR **(B, C, E, F)**. Image adjusted from (20). For comparison, T2 signal changes in rodent brain stem motor nuclei are shown in (21–23).

A neuroscience subfield with particularly low bench-to-bedside translation and only exiguous therapeutic options are motor neuron diseases (MND), including entities such as amyotrophic lateral sclerosis (ALS) (4, 14, 15). In these mostly fatal diseases, magnetic resonance imaging (MRI) has become among the most important paraclinical tools for diagnostic workup (16–19). Although unspecific to MND; MRI can present with certain patterns of brain and spinal cord atrophy as well as signal changes in the corticospinal tract and motor cortex (Figure 1).

A variety of MND animal models are used for pathomechanistic investigations of these disorders, most prominently transgenic rodents with mutations in the SOD1 gene, thus mimicking familial ALS (24). However, it is insufficiently understood how well these animal models mimic human MND imaging phenotypes, i.e., what is external validity of these animal models in the neuroimaging domain? Improved understanding of the external validity of these animal models would not only benefit researchers using these models to assess putative drug candidates for MND, but it would also help to implement refinement strategies from the 3R—reduce, replace, refine—within the field (13, 25).

Thus, based on this shortcoming, we here aim at assessing the external validity of motor neuron disease animal models by systematically summarizing MRI features of MND animal models, and to compare these features with human MRI phenotypes. We focus our analysis on structural MRI as used in the clinical routine for MND diagnostic work-up. This study complements a recently published systematic review on structural neuroimaging findings in human MND (20).

## 2. Methods

### 2.1. Protocol registration

We registered a prospective study protocol in the International Prospective Register of Systematic Reviews (PROSPERO, CRD42022373146, https://www.crd.york.ac.uk/PROSPERO/) and used the Preferred Reporting Items for Systematic Reviews and Meta-Analysis (PRISMA) guidelines for reporting (26).

### 2.2. Search strategy

We searched PubMed and Ovid EMBASE for relevant publications from inception up to December 19, 2022. See Supplementary Table 1 for the search strings in each of these databases.

### 2.3. Inclusion and exclusion criteria

We included original publications that reported on any structural brain or spinal cord MRI outcome in MND animal models. Conference abstracts, non-English articles, and publications which reiterated previously reported quantitative data were excluded. Reviews were excluded but retained as potential sources for additional records. Reference lists of these reviews were screened for additional eligible publications.

### 2.4. Study selection and data extraction

Titles and abstracts of studies were screened for their relevance in the web-based application Rayyan (27) by two independent reviewers followed by full-text screening. From eligible full texts, the following data was extracted by two independent reviewers: title, authors, publication year, journal, MND model, number of animals in the treatment and control groups, MRI static magnetic field strength, and main findings related to structural neuroimaging.

### 2.5. Quality assessment

Risk of bias was assessed against a 3-item checklist according to the consensus statement for good laboratory practice in the modeling of stroke (sample size calculations provided, reporting of animal welfare, statement of a potential conflict of interest) (28), as well as four items on reporting any measure of randomization or blinding (29).

## 3. Results

### 3.1. General study characteristics

#### 3.1.1. Eligible publications

**Figure 2:**
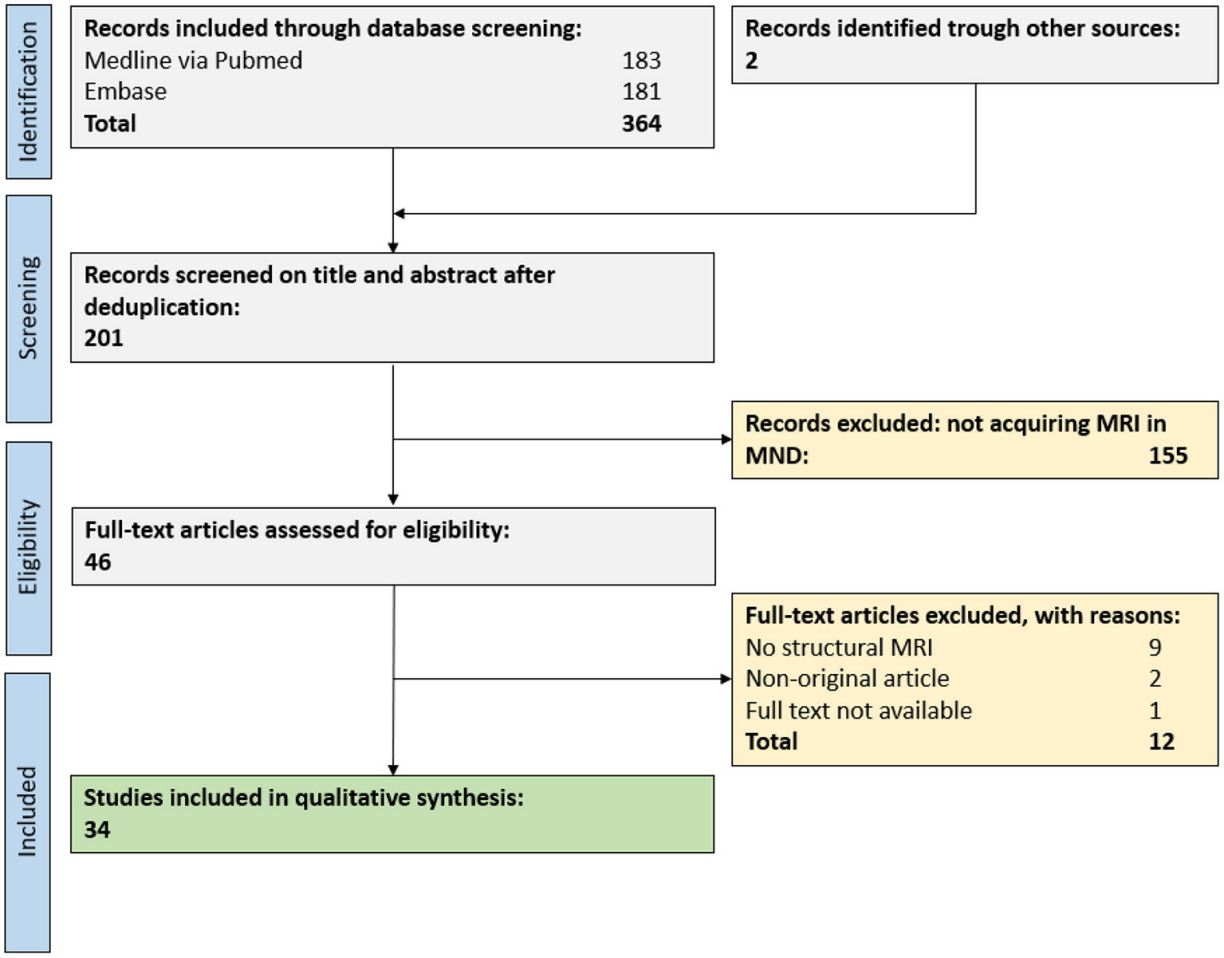
PRISMA flow chart for study inclusion. A total of 34 publications were eligible for the qualitative synthesis. MND, motor neuron disease; MRI, magnetic resonance imaging.

In total, 364 publications were retrieved from our database search, and an additional 2 publications from reference lists of reviews on related topics. After abstract and title screening, 46 publications were eligible for full-text search. After screening the full text of these records, 34 publications (17% of deduplicated references) were included for the qualitative synthesis (Figure 2).

#### 3.1.2. Experimental parameters of eligible publications

The most frequently used MND animal model was the *SOD1*^*G*93*A*^ transgenic model, mimicking familial ALS (26 publications, 76%, we will refer to these models as ALS animal models in the remainder of the manuscript). The B6SJL-Tg(SOD1^G93A^)1Gur/J was the most commonly used mutant (15 publications, 58%), the B6.Cg-Tg(SOD1^G93A^)1Gur/J was only used in one publication, the remaining publications did not further specify the mutant.

Only mice and rats were used in the eligible publications (30 [88%] and 4, [12%], respectively). The employed static magnetic field strengths ranged from 1.5T to 17.6T, with most publications employing 7T (16, 47%). The median sample size of animals was 10 and 5.5 animals for the experimental and control groups, respectively (interquartile range, IQR [7–21.75] and [0.75–7.75], respectively). Four publications did not report the number of used animals.

Seven publications (21%) tested a therapeutic intervention for MND, among them mostly stem cell-based approaches (4 publications, 12%) (21, 30–32). One study each investigated liposomal encapsulated glucocorticoid (33), davunetide (an intranasal neuropeptide therapy) (34), and deferiprone (an iron chelator) (35).

More detailed data on experimental parameters can be found in Supplementary Table 2.

#### 3.1.3. Risk of bias assessment

Most publications showed a low risk of bias in the animal welfare (reported by 29/34 publications, 85%) and conflict of interest domain (19/34, 56%). Yet only few publications reported randomization (7/34, 21%), blinding (6/34, 18%) or sample size calculations for their study (3/34, 9%) (Supplementary Table 3).

### 3.2. Neuroimaging findings in motor neuron disease animal models

#### 3.2.1. Atrophy of brain and spinal cord

Neuroimaging has consistently shown local central nervous system (CNS) tissue volume loss in MND animal models. Yet the affected anatomical CNS regions show a high degree of variability between reports. 1-year old mice overexpressing both *APP* and *SOD1* mutations exhibited gray matter atrophy, most pronounced in the hippocampi as well as in entorhinal and cingulate cortices (36). In contrast, mice only overexpressing SOD1 exhibited atrophy specifically in cortical regions (cingulate, retrosplenial, and temporoparietal cortex) but not in the hippocampi (36). A loss in motor cortex volume has also been observed in the murine *SOD1*^*G*93*A*^ model at postnatal day 100 (37). However, such motor cortex atrophy has not been consistent in other study using mice of similar age (38). Along these lines, a report using the *TARDBP*^*Q*331*K*^ transgenic mouse strain, i.e., a model for ALS-FTD, found a more prominent atrophy in the entorhinal cortex compared to the motor cortex (39). Mice fed with cycad toxins (resulting in motor neuron loss) show lower volumes in the substantia nigra, striatum, basal nucleus/internal capsule, and olfactory bulb (40). A more recent study using a conditional TDP-43 mouse model found progressive volume loss of the gray matter in the olfactory bulb, frontal association cortices, lateral and dorsolateral orbital cortices, agranular insular cortices, globus pallidus, hippocampi, dorsal subiculum, secondary visual cortices, as well as in the cerebellum (41). Finally, several studies described atrophy of brain stem nuclei (42), particularly of motor nuclei, e.g., trigeminal, facial, and hypoglossal nuclei (34, 38).

Spinal cord volume loss has been observed in the murine *SOD1*^*G*93*A*^ model (37, 43), but also in the cycad toxin animal model (40).

#### 3.2.2. Signal changes of brain and spinal cord

T2w hyperintensities have been described in rodent ALS models in the brain stem (21–23, 44, 45). These hyperintensities seem to parallel or even precede first behavioral ALS symptoms (46, 47). Histopathological correlations found associated vacuolar degeneration (23, 45–49) as well as micro- and astroglial activation (42). Interestingly, magnetic resonance microscopy was able to also detect hyperintensities in the ventral motor tracts within the murine spinal cord (50). Higher T2 values, mainly in the ventral portions of the spinal cord, have also been observed using conventional sequences at 7T (51).

One study found iron accumulation in the cervical spinal cord (based on T2^*^ contrast), that, however, disappeared with progressing disease (37). Iron changes have also been observed in the medulla oblongata and motor cortex (35).

#### 3.2.3. Contrast enhancement patterns

Overt breakdown of the blood-brain barrier adjacent to lateral ventricles and in the hippocampal region was described in a rat ALS model (22). Such breakdown of the BBB was consistent in another study which also employed Ultrasmall superparamagnetic iron oxide (USPIO) enhanced MRI (52). Here, BBB breakdown was congruent with T cell infiltration. Finally, a study using dynamic contrast-enhanced MRI upon intracisternal injection of gadolinium found altered contrast medium clearance in ALS model mice compared to controls (41).

## 4. Discussion

### 4.1. Main findings

The main objective of this study was to systematically summarize the available evidence on structural CNS MRI features in ALS animal models. Frequent MRI features include brain and spinal cord atrophy, signal changes in brain stem motor nuclei and the motor cortex as well as breakdown of the blood-brain barrier (Table 1). In the following paragraphs, we will compare this phenotype with MRI features of human ALS.

**Table 1:** Synopsis of brain and spinal cord magnetic resonance imaging findings in amyotrophic lateral sclerosis (ALS) animal models.

**MRI phenotype in ALS rodent models**	**MRI phenotype in human ALS**
**Atrophy of brain and spinal cord**	
**Cortical gray matter**	
Entorhinal (39), cingulate, retrosplenial, temporoparietal (36), motor (37), frontal association, lateral/dorsolateral orbital, agranular insular, and secondary visual cortices (41). No motor cortex atrophy (38)	Motor cortex (53–56); pre- and postcentral gyrus (57). No cortical thinning (58–62)
**Subcortical gray matter**	
Hippocampi (39), substantia nigra, striatum, and basal nucleus (40, 41) as well as brain stem motor nuclei (34, 38, 42)	Hippocampi (57, 58, 63, 64), thalamus (65–67), caudate nucleus, putamen, amygdala (68), and basal ganglia (69). No subcortical volume loss (58–61)
**White matter structures**	
Internal capsule (41)	Overall white matter (70); corpus callosum (69, 71)
**Other brain structures**	
Olfactory bulb (40, 41); cerebellum (41)	Total brain volume (62, 72); cerebellum (73, 74). No cerebellar atrophy (75)
**Spinal cord**	
Spinal cord atrophy (37, 40, 43)	Spinal cord atrophy (63, 76)
**Signal changes of brain and spinal cord**	
**T2 hyperintensities**	
T2 hyperintensities in the brain stem (21–23, 44, 45) and ventral motor tracts of the spinal cord (50, 51)	CST hyperintensity in T2w-FLAIR, but also T2w, PDw, T2*w (77, 78)
**Iron accumulation/motor cortex hypointensity**	
Iron accumulation in the cervical spinal cord (37), medulla oblongata, and motor cortex (35)	Motor cortex hypointensity (motor band sign) on T2w, T2*w, T2w-FLAIR, or SWI (79–82). Iron deposition in deep subcortical gray matter structures (83)
**Contrast enhancement patterns**	
Blood-brain barrier breakdown adjacent to lateral ventricles and in the hippocampal region (37, 47). Altered CSF gadolinium clearance (33)	No imaging data

Comparing magnetic resonance imaging (MRI) findings between amyotrophic lateral sclerosis (ALS) animal models and human ALS. Most commonly reported MRI findings in ALS animal models are brain and spinal cord volume loss, T2 and T2^*^ signal changes as well as contrast-enhancement indicating breakdown of the blood-brain barrier.

ALS, amyotrophic lateral sclerosis; CSF, cerebrospinal fluid; CST, corticospinal tract; FLAIR, fluid-attenuated inversion recovery; FTD, frontotemporal dementia; MND, motor neuron disease; MRI, magnetic resonance imaging; PDw, proton density-weighted; SWI, susceptibility weighted imaging.

### 4.2. Findings in the context of existing evidence

Based on the findings of our systematic review, ALS animal models seem to feature several imaging signs reminiscent of human ALS (Table 1). Among these features is the volume loss of CNS structures with progressive disease. Atrophy in both the motor cortex (37) and the spinal cord (37, 40, 43) has been reported in ALS animal models, similar to the human imaging phenotype (20, 53, 54), which could correspond to the underlying decline of the upper and lower motor neurons (14). These similarities between the human and animal imaging phenotype are particularly interesting since most eligible animal studies used the G93A-SOD1 model thus mimicking familial ALS, a rare clinical phenotype constituting around 10% of ALS patients. It is also noteworthy that, similar to the human population (20), a wide and not always consistent array of CNS structures have been reported to be affected by volume loss in animal models. For example, motor cortex atrophy has not been consistently shown in ALS animal models (38). It is likely that different methodological approaches for the quantification of atrophy patterns between animal studies is in part responsible for these inconsistencies: This has been emphasized by a human study in ALS-FTD patients which found variable atrophy patterns when comparing different software to assess cortical volumes (FSL, FreeSurfer, and SPM) (84). Further confounders could be technical parameters such as intra-/inter-scanner variability and physiological factors such as hydration state of animals during imaging [reviewed in (85)].

ALS rodent models can present with T2 signal changes in the CNS, potentially corresponding to axonal degeneration (23). In rodents, these signal alterations seem to commonly affect brain stem motor nuclei (21, 22). In ALS patients, T2 signal changes are also commonly observed (20, 77), albeit at different locations, i.e., mostly along the corticospinal tract (Figure 1).

Abnormal iron deposition in the motor cortex and spinal cord has been reported by some rodent ALS studies, measured by T2^*^-based MRI approaches (35, 37). Although respective publications did not include pictorial examples of iron deposition within the motor cortex, this feature could correspond to the “motor band sign” (linear motor cortex hypointensity) which is commonly observed in the motor cortex of ALS patients on T2^*^-based sequences (Figure 1). In ALS, these signal drops seem to correspond to astro- and microglia iron deposition within deep layers of the motor cortex (86).

One imaging feature which seems more specific to rodent ALS models is breakdown of the blood-brain barrier, as visualized by gadolinium enhancement in periventricular and hippocampal regions (22). However, although gadolinium enhancement is not observed in the clinical setting in ALS, several lines of evidence demonstrate damage to the blood–brain and blood-spinal cord barrier in ALS [reviewed in (87)]. Such vascular changes seem to include alterations of tight junction proteins (88) and can be observed already early in the disease process (89). Structural MRI features of preclinical ALS models are summarized in Table 1, alongside with MRI features of human ALS.

### 4.3. Limitations

To assess the external validity of ALS animal models, we focused our analysis on structural brain and spinal cord MRI features. However, other disease aspects such as patterns of physical disability or also more advanced MRI methods like diffusion-tensor imaging, which are able to more specifically reflect pathogenic disease processes, might enable a more comprehensive comparison between experimental and human phenotypes.

A genuine limitation of this systematic review is that only a limited number of studies employing MRI in ALS animal models was eligible. As a result, it is difficult to map imaging phenotypes of less commonly used ALS models such as cycad toxins or wobbler mice or even for different SOD1^G93A^ mutants. It is possible that certain ALS rodent models might mimic specific human imaging phenotypes better than others (36), similarly to the situation in experimental autoimmune encephalomyelitis (EAE)—a commonly used animal model for multiple sclerosis (90).

Finally, although seven of the eligible publications tested a putative therapeutic intervention for ALS, no corresponding human MRI studies could be identified. Correlating the impact of therapeutic interventions on neuroimaging phenotypes between rodent models and humans would further enhance understanding of the translational value of experimental ALS models.

## 5. Conclusions

Our systematic review provides high-grade evidence that preclinical ALS models do show imaging features highly reminiscent of human ALS, including certain brain and spinal cord atrophy patterns and signal changes in motor systems (Table 1). Certain imaging features such as breakdown of the BBB are only partly reflected by these experimental models. Thus, ALS rodent models show a high external validity in the neuroimaging domain. This contrasts the high attrition of drugs in clinical ALS trials which have shown promising results in ALS animal models; and this raises concerns that a mere phenotypic comparability between experimental models and corresponding human diseases does not necessarily render an animal model appropriate for drug development. These findings emphasize a careful application of these model systems for ALS drug development thereby benefiting refinement of animal experiments.

## Data availability statement

The original contributions presented in the study are included in the article/Supplementary material, further inquiries can be directed to the corresponding author.

## Author contributions

CZ, TG, and BVI conceived the study. AC, WZ, CZ, and BVI performed the literature review and data extraction. BVI wrote the manuscript. All authors provided critical input on the manuscript. All authors contributed to the article and approved the submitted version.

## References

[1] Wong CH Siah KW Lo AW. Estimation of clinical trial success rates and related parameters. Biostatistics. (2019) 20:273–86. 10.1093/biostatistics/kxx069 29394327 PMC6409418

[2] I Kola I Landis J. Can the pharmaceutical industry reduce attrition rates? Nat Rev Drug Disc. (2004) 3:711. 10.1038/nrd1470 15286737

[3] Bespalov A Steckler T Altevogt B Koustova E Skolnick P Deaver D et al. Failed trials for central nervous system disorders do not necessarily invalidate preclinical models and drug targets. Nat Rev Drug Disc. (2016) 15:516–516. 10.1038/nrd.2016.88 27312728

[4] Scott S Kranz JE Cole J Lincecum JM Thompson K Kelly N et al. Design, power, and interpretation of studies in the standard murine model of ALS. Amyotrophic Lateral Scleros. (2008) 9:4–15. 10.1080/17482960701856300 18273714

[5] Waring MJ Arrowsmith J Leach AR Leeson PD Mandrell S Owen RM . An analysis of the attrition of drug candidates from four major pharmaceutical companies. Nat Rev Drug Disc. (2015) 14:475–86. 10.1038/nrd4609 26091267

[6] Honkala A Malhotra SV Kummar S Junttila MR. Harnessing the predictive power of preclinical models for oncology drug development. Nat Rev Drug Disc. (2021) 3:1–16. 10.1038/s41573-021-00301-6 34702990

[7] Bespalov A Bernard R Gilis A Gerlach B Guillen J Castagne V . Introduction to the EQIPD quality system. Elife. (2021) 10:12. 10.7554/eLife.63294.sa2 PMC8184207 34028353

[8] Ritskes–Hoitinga M van Luijk J. How can systematic reviews teach us more about the implementation of the 3Rs and animal welfare? Animals. (2019) 9:1163. 10.3390/ani9121163 31861205 PMC6941037

[9] Ioannidis JP Greenland S Hlatky MA Khoury MJ Macleod MR Moher D . Increasing value and reducing waste in research design, conduct, and analysis. Lancet. (2014) 383:166–75. 10.1016/S0140-6736(13)62227-8 24411645 PMC4697939

[10] Vollert J Schenker E Macleod M Bespalov A Wuerbel H Michel M . Systematic review of guidelines for internal validity in the design, conduct and analysis of preclinical biomedical experiments involving laboratory animals. BMJ open science. (2020) 4:e100046. 10.1136/bmjos-2019-100046 35047688 PMC8647591

[11] Van der Worp HB Howells DW Sena ES Porritt MJ Rewell S O'Collins V . Macleod. Can animal models of disease reliably inform human studies? PLoS Med. (2010) 7:e1000245. 10.1371/journal.pmed.1000245 20361020 PMC2846855

[12] Ferreira G Veening–Griffioen DH Boon WP Moors EH Gispen–de Wied CC Schellekens H . A standardised framework to identify optimal animal models for efficacy assessment in drug development. PLoS ONE. (2019) 14:e0218014. 10.1371/journal.pone.0218014 31194784 PMC6563989

[13] Ferreira GS Veening–Griffioen DH Boon WP Moors EH van Meer PJ. Levelling the translational gap for animal to human efficacy data. Animals. (2020) 10:1199. 10.3390/ani10071199 32679706 PMC7401509

[14] Kiernan MC Vucic S Cheah BC Turner MR Eisen A Hardiman O . Amyotrophic lateral sclerosis. Nat Rev Dis Prim. (2017) 3:17071. 10.1038/nrdp.2017.72 28980624

[15] Rosenfeld J Strong MJ. Challenges in the understanding and treatment of amyotrophic lateral sclerosis/motor neuron disease. Neurotherapeutics. (2015) 12:317–25. 10.1007/s13311-014-0332-8 25572957 PMC4404444

[16] Goodin DS Rowley HA Olney RK. Magnetic resonance imaging in amyotrophic lateral sclerosis. Neurol Res Int. (2012) 2012:165. 10.1002/ana.410230424 3382182

[17] Kassubek J Pagani M. Imaging in amyotrophic lateral sclerosis: MRI and PET. Curr Opin Neurol. (2019) 32:740–6. 10.1097/WCO.0000000000000728 31335337

[18] Kassubek J Müller HP. Computer–based magnetic resonance imaging as a tool in clinical diagnosis in neurodegenerative diseases. Expert Rev Neurother. (2016) 16:295–306. 10.1586/14737175.2016.1146590 26807776

[19] Bede P Hardiman O. Lessons of ALS imaging: pitfalls and future directions—a critical review. NeuroImage: Clinical. (2014) 4:436–43. 10.1016/j.nicl.2014.02.011 24624329 PMC3950559

[20] Zejlon C Nakhostin D Winklhofer S Pangalu A Kulcsar Z Lewandowski S . Structural magnetic resonance imaging findings and histopathological correlations in motor neuron diseases—A systematic review and meta–analysis. Front Neurol. (2022) 13:947347. 10.3389/fneur.2022.947347 36110394 PMC9468579

[21] Bontempi P Busato A Bonafede R Schiaffino L Scambi I Sbarbati A . MRI reveals therapeutical efficacy of stem cells: an experimental study on the SOD1(G93A) animal model. Mag Res Med. (2018) 79:459–69. 10.1002/mrm.26685 28370153

[22] Andjus PR Bataveljić D Vanhoutte G Mitrecic D Pizzolante F Djogo N . In vivo morphological changes in animal models of amyotrophic lateral sclerosis and Alzheimer's–like disease: MRI approach. Anat Record. (2009) 292:1882–92. 10.1002/ar.20995 19943341

[23] Zang DW Yang Q Wang HX Egan G Lopes EC Cheema SS. Magnetic resonance imaging reveals neuronal degeneration in the brainstem of the superoxide dismutase 1 transgenic mouse model of amyotrophic lateral sclerosis. Eur J Neurosci. (2004) 20:1745–51. 10.1111/j.1460-9568.2004.03648.x 15379995

[24] Philips T Rothstein JD. Rodent Models of Amyotrophic Lateral Sclerosis. Curr. Prot. Pharmacol. (2015) 69:5.67.1–5.67.21. 10.1002/0471141755.ph0567s69 26344214 PMC4562058

[25] Macleod M Mohan S. Reproducibility and rigor in animal–based research. ILAR J. (2019) 60:17–23. 10.1093/ilar/ilz015 31687758 PMC7275809

[26] Moher D Shamseer L Clarke M Ghersi D Liberati A Petticrew M . Preferred reporting items for systematic review and meta–analysis protocols. (PRISMA–P) 2015 statement. Syst Rev. (2015) 4:1. 10.1186/2046-4053-4-1 25554246 PMC4320440

[27] Ouzzani M Hammady H Fedorowicz Z Elmagarmid A. Rayyan—a web and mobile app for systematic reviews. Syst Rev. (2016) 5:210. 10.1186/s13643-016-0384-4 27919275 PMC5139140

[28] Macleod MR Fisher M. O'collins V, Sena ES, Dirnagl U, Bath PM. Good laboratory practice: preventing introduction of bias at the bench. J Int Soc Cereb Blood Flow Metabol. (2009) 29:221–3. 10.1038/jcbfm.2008.101 18797473 PMC2729492

[29] Hooijmans CR Hlavica M Schuler FA Good N Good A Baumgartner L . Remyelination promoting therapies in multiple sclerosis animal models: a systematic review and meta–analysis. Sci Rep. (2019) 9:822. 10.1038/s41598-018-35734-4 30696832 PMC6351564

[30] Bigini P Diana V Barbera S Fumagalli E Micotti E Sitia L . Longitudinal tracking of human fetal cells labeled with super paramagnetic iron oxide nanoparticles in the brain of mice with motor neuron disease. PLoS ONE. (2012) 7:e32326. 10.1371/journal.pone.0032326 22384217 PMC3288077

[31] Bonafede R Turano E Scambi I Busato A Bontempi P Virla F . ASC–Exosomes ameliorate the disease progression in SOD1(G93A) murine model underlining their potential therapeutic use in human ALS. Int J Mol Sci. (2020) 21:15. 10.3390/ijms21103651 32455791 PMC7279464

[32] Canzi L Castellaneta V Navone S Nava S Dossena M Zucca I . Human skeletal muscle stem cell antiinflammatory activity ameliorates clinical outcome in amyotrophic lateral sclerosis models. Mol Med. (2012) 18:401–11. 10.2119/molmed.2011.00123 22076467 PMC3356418

[33] Evans MC Gaillard PJ de Boer M Appeldoorn C Dorland R Sibson NR . CNS–targeted glucocorticoid reduces pathology in mouse model of amyotrophic lateral sclerosis. Acta Neuropathol Commun. (2014) 2:66. 10.1186/2051-5960-2-66 24923195 PMC4229735

[34] Jouroukhin Y Ostritsky R Assaf Y Pelled G Giladi E Gozes INAP. (davunetide) modifies disease progression in a mouse model of severe neurodegeneration: protection against impairments in axonal transport. Neurobiol Dis. (2013) 56:79–94. 10.1016/j.nbd.2013.04.012 23631872

[35] Moreau C Danel V Devedjian JC Grolez G Timmerman K Laloux C . Could Conservative iron chelation lead to neuroprotection in amyotrophic lateral sclerosis? Antioxid Redox Signal. (2018) 29:742–8. 10.1089/ars.2017.7493 29287521 PMC6067092

[36] Borg J Chereul E. Differential MRI patterns of brain atrophy in double or single transgenic mice for APP and/or SOD. J Neurosci Res. (2008) 86:3275–84. 10.1002/jnr.21778 18646206

[37] Grolez G Kyheng M Lopes R Moreau C Timmerman K Auger F . MRI of the cervical spinal cord predicts respiratory dysfunction in ALS. Sci Rep. (2018) 8:1828. 10.1038/s41598-018-19938-2 29379040 PMC5789036

[38] Marcuzzo S Zucca I Mastropietro A de Rosbo NK Cavalcante P Tartari S . Hind limb muscle atrophy precedes cerebral neuronal degeneration in G93A–SOD1 mouse model of amyotrophic lateral sclerosis: a longitudinal MRI study. Exp Neurol. (2011) 231:30–7. 10.1016/j.expneurol.2011.05.007 21620832

[39] White MA Lin Z Kim E Henstridge CM Pena Altamira E Hunt CK . Sarm1 deletion suppresses TDP−43–linked motor neuron degeneration and cortical spine loss. Acta Neuropathol Commun. (2019) 7:166. 10.1186/s40478-019-0800-9 31661035 PMC6819591

[40] Wilson JM Petrik MS Grant SC Blackband SJ Lai J Shaw CA. Quantitative measurement of neurodegeneration in an ALS–PDC model using MR microscopy. Neuroimage. (2004) 23:336–43. 10.1016/j.neuroimage.2004.05.026 15325381

[41] Zamani A Walker AK Rollo B Ayers KL Farah R O'Brien TJ . Impaired glymphatic function in the early stages of disease in a TDP−43 mouse model of amyotrophic lateral sclerosis. Transl Neurodegener. (2022) 11:17. 10.1186/s40035-022-00291-4 35287738 PMC8922788

[42] Evans MC Serres S Khrapitchev AA Stolp HB Anthony DC Talbot K . T_2_-weighted MRI detects presymptomatic pathology in the SOD1 mouse model of ALS. J Cereb Blood Flow Metab. (2014) 34:785–93. 10.1038/jcbfm.2014.19 24496176 PMC4013759

[43] Marcuzzo S Bonanno S Figini M Scotti A Zucca I Minati L . A longitudinal DTI and histological study of the spinal cord reveals early pathological alterations in G93A–SOD1 mouse model of amyotrophic lateral sclerosis. Exp Neurol. (2017) 293:43–52. 10.1016/j.expneurol.2017.03.018 28351750

[44] Grant RA Sharp PS Kennerley AJ Berwick J Grierson A Ramesh T . Abnormalities in whisking behaviour are associated with lesions in brain stem nuclei in a mouse model of amyotrophic lateral sclerosis. Behav Brain Res. (2014) 259:274–83. 10.1016/j.bbr.2013.11.002 24239688

[45] Bataveljić D Djogo N Zupunski L Bajić A Nicaise C Pochet R . Live monitoring of brain damage in the rat model of amyotrophic lateral sclerosis. Gen Physiol Biophys. (2009) 28:212–8. 19893103

[46] Angenstein F Niessen HG Goldschmidt J Vielhaber S Ludolph AC Scheich H. Age–dependent changes in MRI of motor brain stem nuclei in a mouse model of ALS. Neuroreport. (2004) 15:2271–4. 10.1097/00001756-200410050-00026 15371748

[47] Majchrzak M Drela K Andrzejewska A Rogujski P Figurska S Fiedorowicz M . SOD1/Rag2 mice with low copy number of SOD1 gene as a new long–living immunodeficient model of ALS. Sci Rep. (2019) 9:799. 10.1038/s41598-018-37235-w 30692571 PMC6349855

[48] Bucher S Braunstein KE Niessen HG Kaulisch T Neumaier M Boeckers TM . Vacuolization correlates with spin–spin relaxation time in motor brainstem nuclei and behavioural tests in the transgenic G93A–SOD1 mouse model of ALS. Eur J Neurosci. (2007) 26:1895–901. 10.1111/j.1460-9568.2007.05831.x 17868365

[49] Caron I Micotti E Paladini A Merlino G Plebani L Forloni G . Comparative magnetic resonance imaging and histopathological correlates in Two SOD1 transgenic mouse models of amyotrophic lateral sclerosis. PLoS ONE. (2015) 10:e0132159. 10.1371/journal.pone.0132159 26132656 PMC4488470

[50] Cowin GJ Butler TJ Kurniawan ND Watson C Wallace RH. Magnetic resonance microimaging of the spinal cord in the SOD1 mouse model of amyotrophic lateral sclerosis detects motor nerve root degeneration. Neuroimage. (2011) 58:69–74. 10.1016/j.neuroimage.2011.06.003 21689764

[51] Niessen HG Angenstein F Sander K Kunz WS Teuchert M Ludolph AC . In vivo quantification of spinal and bulbar motor neuron degeneration in the G93A–SOD1 transgenic mouse model of ALS by T2 relaxation time and apparent diffusion coefficient. Exp Neurol. (2006) 201:293–300. 10.1016/j.expneurol.2006.04.007 16740261

[52] Bataveljić D Stamenković S Bačić G Andjus P. Imaging cellular markers of neuroinflammation in the brain of the rat model of amyotrophic lateral sclerosis. Acta Physiol Hung. (2011) 98:27–31. 10.1556/APhysiol.98.2011.1.4 21388928

[53] Verstraete E Veldink JH Hendrikse J Schelhaas HJ Van Den Heuvel MP . Structural MRI reveals cortical thinning in amyotrophic lateral sclerosis. J Neurol Neurosurg Psychiatry. (2012) 83:383–8. 10.1136/jnnp-2011-300909 21965521

[54] Menke RA Proudfoot M Talbot K Turner MR. The two–year progression of structural and functional cerebral MRI in amyotrophic lateral sclerosis. NeuroImage Clin. (2018) 17:953–61. 10.1016/j.nicl.2017.12.025 29321969 PMC5752097

[55] Butman JA Floeter MK. Decreased thickness of primary motor cortex in primary lateral sclerosis. Ajnr: Am J Neuroradiol. (2007) 28:87–91. 17213431 PMC8134097

[56] Schuster C Kasper E Machts J Bittner D Kaufmann J Benecke R . Longitudinal course of cortical thickness decline in amyotrophic lateral sclerosis. J Neurol. (2014) 261:1871–80. 10.1007/s00415-014-7426-4 25022938

[57] Cosottini M Pesaresi I Piazza S Diciotti S Cecchi P Fabbri S . Structural and functional evaluation of cortical motor areas in Amyotrophic Lateral Sclerosis. Exp Neurol. (2012) 234:169–80. 10.1016/j.expneurol.2011.12.024 22226599

[58] Acosta–Cabronero J Machts J Schreiber S Abdulla S Kollewe K Petri S . Quantitative susceptibility MRI to detect brain iron in amyotrophic lateral sclerosis. Radiology. (2018) 289:195–203. 10.1148/radiol.2018180112 30040038 PMC6166868

[59] Agosta F Spinelli EG Riva N Fontana A Basaia S Canu E . Survival prediction models in motor neuron disease. Eur J Neurol. (2019) 26:1143–52. 10.1111/ene.13957 30920076

[60] Cardenas–Blanco A Machts J Acosta–Cabronero J Kaufmann J Abdulla S Kollewe K . Structural and diffusion imaging versus clinical assessment to monitor amyotrophic lateral sclerosis. NeuroImage Clin. (2016) 11:408–414. 10.1016/j.nicl.2016.03.011 27104135 PMC4827722

[61] Duning T Schiffbauer H Warnecke T Mohammadi S Floel A Kolpatzik K . G–CSF prevents the progression of structural disintegration of white matter tracts in amyotrophic lateral sclerosis: a pilot trial. PLoS ONE. (2011) 6:e17770. 10.1371/journal.pone.0017770 21423758 PMC3056779

[62] Ellis CM Suckling J Amaro E Jr Bullmore ET Simmons A Williams SC . Volumetric analysis reveals corticospinal tract degeneration and extramotor involvement in ALS. Neurology. (2001) 57:1571–8. 10.1212/WNL.57.9.1571 11706094

[63] Piaggio N Pardini M Roccatagliata L Scialò C Cabona C Bonzano L . Cord cross–sectional area at foramen magnum as a correlate of disability in amyotrophic lateral sclerosis. Eur Radiol Exp. (2018) 2:13. 10.1186/s41747-018-0045-6 29984352 PMC6015117

[64] Thorns J Jansma H Peschel T Grosskreutz J Mohammadi B Dengler R . Extent of cortical involvement in amyotrophic lateral sclerosis–an analysis based on cortical thickness. BMC Neurol. (2013) 13:148. 10.1186/1471-2377-13-148 24138960 PMC3853794

[65] Buhour MS Doidy F Mondou A Pélerin A Carluer L Eustache F . Voxel–based mapping of grey matter volume and glucose metabolism profiles in amyotrophic lateral sclerosis. EJNMMI Res. (2017) 7:21. 10.1186/s13550-017-0267-2 28266002 PMC5339262

[66] Agosta F Basaia S Trojsi F Riva N Cividini C Femiano C . Structrural and functional organization of the brain connectome in patients with different motor neuron disease: a multicenter study. Neurology. (2019) 92:3. 10.1212/WNL.0000000000010731 PMC7682834 32913015

[67] Bocchetta M Gordon E Cardoso MJ Modat M Ourselin S Warren JD . Thalamic atrophy in frontotemporal dementia — Not just a C9orf72 problem. NeuroImage: Clinical. (2018) 18:675–81. 10.1016/j.nicl.2018.02.019 29876259 PMC5988457

[68] Pallebage–Gamarallage M Foxley S Menke RA Huszar IN Jenkinson M Tendler BC . Dissecting the pathobiology of altered MRI signal in amyotrophic lateral sclerosis: A post mortem whole brain sampling strategy for the integration of ultra–high–field MRI and quantitative neuropathology. BMC Neurosci. (2018) 19:11. 10.1186/s12868-018-0416-1 29529995 PMC5848544

[69] Menke RA Körner S Filippini N Douaud G Knight S Talbot K . Widespread grey matter pathology dominates the longitudinal cerebral MRI and clinical landscape of amyotrophic lateral sclerosis. Brain. (2014) 137:2546–55. 10.1093/brain/awu162 24951638 PMC4132644

[70] Senda J Kato S Kaga T Ito M Atsuta N Nakamura T . Progressive and widespread brain damage in ALS: MRI voxel–based morphometry and diffusion tensor imaging study. Amyotrophic Lat Scler. (2011) 12:59–69. 10.3109/17482968.2010.517850 21271792

[71] Müller HP Dreyhaupt J Roselli F Schlecht M Ludolph AC Huppertz HJ . Focal alterations of the callosal area III in primary lateral sclerosis: an MRI planimetry and texture analysis. NeuroImage Clin. (2020) 26:102223. 10.1016/j.nicl.2020.102223 32114375 PMC7049663

[72] Mahoney CJ Downey LE Ridgway GR Beck J Clegg S Blair M . Longitudinal neuroimaging and neuropsychological profiles of frontotemporal dementia with C9ORF72 expansions. Alzheimer's Res Therapy. (2012) 4:41. 10.1186/alzrt144 23006986 PMC3580398

[73] Agosta F Ferraro PM Riva N Spinelli EG Domi T Carrera P . Structural and functional brain signatures of C9orf72 in motor neuron disease. Neurobiol Aging. (2017) 57:206–19. 10.1016/j.neurobiolaging.2017.05.024 28666709

[74] Mahoney CJ Beck J Rohrer JD Lashley T Mok K Shakespeare T . Frontotemporal dementia with the C9ORF72 hexanucleotide repeat expansion: clinical, neuroanatomical and neuropathological features. Brain. (2012) 135:736–50. 10.1093/brain/awr361 22366791 PMC3286330

[75] Consonni M Dalla Bella E Nigri A Pinardi C Demichelis G Porcu L . Cognitive syndromes and C9orf72 mutation are not related to cerebellar degeneration in amyotrophic lateral sclerosis. Front Neurosci. (2019) 13:25. 10.3389/fnins.2019.00440 31133784 PMC6524613

[76] El Mendili MM Cohen–Adad J Pelegrini–Issac M Rossignol S Morizot–Koutlidis R Marchand–Pauvert V . Multi–parametric spinal cord MRI as potential progression marker in amyotrophic lateral sclerosis. PLoS ONE. (2014) 9:e95516. 10.1371/journal.pone.0095516 24755826 PMC3995720

[77] Fabes J Matthews L Filippini N Talbot K Jenkinson M Turner MR. Quantitative FLAIR MRI in amyotrophic lateral sclerosis. Acad Radiol. (2017) 24:1187–94. 10.1016/j.acra.2017.04.008 28572001 PMC5605225

[78] Goodin DS Rowley HA Olney RK. Magnetic resonance imaging in amyotrophic lateral sclerosis. Ann Neurol. (1988) 23:418–20. 10.1002/ana.410230424 3382182

[79] Boll MC Meléndez OR Rios C Zenil JM de Alba Y. Is the hypointensity in motor cortex the hallmark of amyotrophic lateral sclerosis? Can J Neurol Sci. (2019) 46:166–73. 10.1017/cjn.2018.382 30724145

[80] Hecht MJ Fellner C Schmid A Neundörfer B Fellner FA. Cortical T2 signal shortening in amyotrophic lateral sclerosis is not due to iron deposits. Neuroradiology. (2005) 47:805–8. 10.1007/s00234-005-1421-5 16175348

[81] Goodin DS Rowley HA Olney RK. Magnetic resonance imaging in amyotrophic lateral sclerosis. Acta Neurol Scand. (2002) 105:395–9. 10.1034/j.1600-0404.2002.01321.x 11982492

[82] Graham JM Papadakis N Evans J Widjaja E Romanowski CA Paley MN . Diffusion tensor imaging for the assessment of upper motor neuron integrity in ALS. Neurology. (2004) 63:2111–9. 10.1212/01.WNL.0000145766.03057.E7 15596758

[83] De Reuck JL Deramecourt V Auger F Durieux N Cordonnier C Devos D . Iron deposits in post–mortem brains of patients with neurodegenerative and cerebrovascular diseases: a semi–quantitative 70 T magnetic resonance imaging study. Eur J Neurol. (2014) 21:1026–31. 10.1111/ene.12432 24698410

[84] Rajagopalan V Pioro EP. Disparate voxel based morphometry. (VBM) results between SPM and FSL softwares in ALS patients with frontotemporal dementia: which VBM results to consider? BMC Neurol. (2015) 15:1–7. 10.1186/s12883-015-0274-8 25879588 PMC4371611

[85] Sastre–Garriga J Pareto D Battaglini M Rocca MA Ciccarelli O Enzinger C . MAGNIMS consensus recommendations on the use of brain and spinal cord atrophy measures in clinical practice. Nature reviews. Neurology. (2020) 16:171–182. 10.1038/s41582-020-0314-x 32094485 PMC7054210

[86] J Kwan JY Jeong SY Van Gelderen P Deng HX Quezado MM Danielian LE . Iron accumulation in deep cortical layers accounts for MRI signal abnormalities in ALS: correlating 7 tesla MRI and pathology. PLoS ONE. (2012) 7:e35241. 10.1371/journal.pone.0035241 22529995 PMC3328441

[87] Sweeney MD Sagare AP Zlokovic BV. Blood–brain barrier breakdown in Alzheimer disease and other neurodegenerative disorders. Nat Rev Neurol. (2018) 14:133. 10.1038/nrneurol.2017.188 29377008 PMC5829048

[88] Zhong Z Deane R Ali Z Parisi M Shapovalov Y O'Banion MK . ALS–causing SOD1 mutants generate vascular changes prior to motor neuron degeneration. Nat Neurosci. (2008) 11:420–2. 10.1038/nn2073 18344992 PMC2895310

[89] Lewandowski SA Nilsson I Fredriksson L Lönnerberg P Muhl L Zeitelhofer M . Presymptomatic activation of the PDGF–CC pathway accelerates onset of ALS neurodegeneration. Acta Neuropathol. (2016) 131:453–64. 10.1007/s00401-015-1520-2 26687981 PMC4839168

[90] Lassmann H Bradl M. Multiple sclerosis: experimental models and reality. Acta Neuropathol. (2016) 3:14. 10.1007/s00401-016-1631-4 27766432 PMC5250666

