# Supplementary material for: Neuroimaging findings in preclinical amyotrophic lateral sclerosis models—How well do they mimic the clinical phenotype? A systematic review
Source: Front Vet Sci. 2023 May 2;10:1135282. doi: 10.3389/fvets.2023.1135282 (PMC10185801; doi:10.3389/fvets.2023.1135282)
Supplement: Supplementary file 1 [file Data_Sheet_1.docx]

**Neuroimaging findings in preclinical amyotrophic lateral sclerosis models – how well do they mimic human amyotrophic lateral sclerosis? A systematic review**

Amelia Elaine Cannon^1^*, Wolfgang Emanuel Zürrer^1^*, Charlotte Zejlon^2^, Zsolt Kulcsar^3^, Sebastian Lewandowski^4^, Fredrik Piehl^4,5^, Tobias Granberg^2,4^, Benjamin Victor Ineichen^1,3,4^

^1^ Center for Reproducible Science, University of Zurich, Zurich, Switzerland.

^2^ Department of Neuroradiology, Karolinska University Hospital, Stockholm, Sweden.

^3^ Department of Neuroradiology, Clinical Neuroscience Center, University Hospital Zurich, University of Zurich, Switzerland.

^4^ Department of Clinical Neuroscience, Karolinska Institutet, Stockholm, Sweden.

^5^ Center of Neurology, Academic Specialist Center, Stockholm Health Services, Stockholm, Sweden.

*Authors contributed equally and share first authorship

**Corresponding author**

Benjamin V. Ineichen: benjaminvictor.ineichen@uzh.ch, OrcID: 0000-0003-1362-4819

University of Zurich, Center for Reproducible Science, Hirschengraben 84, CH-8001 Zurich, Switzerland

**Running head**

MND models translation

**Supplementary data**

**Supplementary Table 1**: Predefined systematic search strings in Medline via PubMed. Last search December 12, 2022. The search retrieved 199 unique publications. An additional two publications were identified through reference lists of eligible articles.

| Animal filter for Pubmed [[1](#_ENREF_1)] and EMBASE [[2](#_ENREF_2)] | ("animal experimentation"[MeSH Terms] OR "models, animal"[MeSH Terms] OR "invertebrates"[MeSH Terms] OR "Animals"[Mesh:noexp] OR "animal population groups"[MeSH Terms] OR "chordata"[MeSH Terms:noexp] OR "chordata, nonvertebrate"[MeSH Terms] OR "vertebrates"[MeSH Terms:noexp] OR "amphibians"[MeSH Terms] OR "birds"[MeSH Terms] OR "fishes"[MeSH Terms] OR "reptiles"[MeSH Terms] OR "mammals"[MeSH Terms:noexp] OR "primates"[MeSH Terms:noexp] OR "artiodactyla"[MeSH Terms] OR "carnivora"[MeSH Terms] OR "cetacea"[MeSH Terms] OR "chiroptera"[MeSH Terms] OR "elephants"[MeSH Terms] OR "hyraxes"[MeSH Terms] OR "insectivora"[MeSH Terms] OR "lagomorpha"[MeSH Terms] OR "marsupialia"[MeSH Terms] OR "monotremata"[MeSH Terms] OR "perissodactyla"[MeSH Terms] OR "rodentia"[MeSH Terms] OR "scandentia"[MeSH Terms] OR "sirenia"[MeSH Terms] OR "xenarthra"[MeSH Terms] OR "haplorhini"[MeSH Terms:noexp] OR "strepsirhini"[MeSH Terms] OR "platyrrhini"[MeSH Terms] OR "tarsii"[MeSH Terms] OR "catarrhini"[MeSH Terms:noexp] OR "cercopithecidae"[MeSH Terms] OR "hylobatidae"[MeSH Terms] OR "hominidae"[MeSH Terms:noexp] OR "gorilla gorilla"[MeSH Terms] OR "pan paniscus"[MeSH Terms] OR "pan troglodytes"[MeSH Terms] OR "pongo pygmaeus"[MeSH Terms]) OR ((animals[tiab] OR animal[tiab] OR mice[Tiab] OR mus[Tiab] OR mouse[Tiab] OR murine[Tiab] OR woodmouse[tiab] OR rats[Tiab] OR rat[Tiab] OR murinae[Tiab] OR muridae[Tiab] OR cottonrat[tiab] OR cottonrats[tiab] OR hamster[tiab] OR hamsters[tiab] OR cricetinae[tiab] OR rodentia[Tiab] OR rodent[Tiab] OR rodents[Tiab] OR pigs[Tiab] OR pig[Tiab] OR swine[tiab] OR swines[tiab] OR piglets[tiab] OR piglet[tiab] OR boar[tiab] OR boars[tiab] OR "sus scrofa"[tiab] OR ferrets[tiab] OR ferret[tiab] OR polecat[tiab] OR polecats[tiab] OR "mustela putorius"[tiab] OR "guinea pigs"[Tiab] OR "guinea pig"[Tiab] OR cavia[Tiab] OR callithrix[Tiab] OR marmoset[Tiab] OR marmosets[Tiab] OR cebuella[Tiab] OR hapale[Tiab] OR octodon[Tiab] OR chinchilla[Tiab] OR chinchillas[Tiab] OR gerbillinae[Tiab] OR gerbil[Tiab] OR gerbils[Tiab] OR jird[Tiab] OR jirds[Tiab] OR merione[Tiab] OR meriones[Tiab] OR rabbits[Tiab] OR rabbit[Tiab] OR hares[Tiab] OR hare[Tiab] OR diptera[Tiab] OR flies[Tiab] OR fly[Tiab] OR dipteral[Tiab] OR drosophila[Tiab] OR drosophilidae[Tiab] OR cats[Tiab] OR cat[Tiab] OR carus[Tiab] OR felis[Tiab] OR nematoda[Tiab] OR nematode[Tiab] OR nematodes[Tiab] OR sipunculida[Tiab] OR dogs[Tiab] OR dog[Tiab] OR canine[Tiab] OR canines[Tiab] OR canis[Tiab] OR sheep[Tiab] OR sheeps[Tiab] OR mouflon[Tiab] OR mouflons[Tiab] OR ovis[Tiab] OR goats[Tiab] OR goat[Tiab] OR capra[Tiab] OR capras[Tiab] OR rupicapra[Tiab] OR rupicapras[Tiab] OR chamois[Tiab] OR haplorhini[Tiab] OR monkey[Tiab] OR monkeys[Tiab] OR anthropoidea[Tiab] OR anthropoids[Tiab] OR saguinus[Tiab] OR tamarin[Tiab] OR tamarins[Tiab] OR leontopithecus[Tiab] OR hominidae[Tiab] OR ape[Tiab] OR apes[Tiab] OR "panpaniscus"[Tiab] OR bonobo[Tiab] OR bonobos[Tiab] OR "pan troglodytes"[Tiab] OR gibbon[Tiab] OR gibbons[Tiab] OR siamang[Tiab] OR siamangs[Tiab] OR nomascus[Tiab] OR symphalangus[Tiab] OR chimpanzee[Tiab] OR chimpanzees[Tiab] OR prosimian[Tiab] OR prosimians[Tiab] OR "bush baby"[Tiab] OR bush babies[Tiab] OR galagos[Tiab] OR galago[Tiab] OR pongidae[Tiab] OR gorilla[Tiab] OR gorillas[Tiab] OR "pongo pygmaeus"[Tiab] OR orangutan[Tiab] OR orangutans[Tiab] OR lemur[Tiab] OR lemurs[Tiab] OR lemuridae[Tiab] OR horse[Tiab] OR horses[Tiab] OR equus[Tiab] OR cow[Tiab] OR calf[Tiab] OR bull[Tiab] OR chicken[Tiab] OR chickens[Tiab] OR gallus[Tiab] OR quail[Tiab] OR bird[Tiab] OR birds[Tiab] OR quails[Tiab] OR poultry[Tiab] OR poultries[Tiab] OR fowl[Tiab] OR fowls[Tiab] OR reptile[Tiab] OR reptilia[Tiab] OR reptiles[Tiab] OR snakes[Tiab] OR snake[Tiab] OR lizard[Tiab] OR lizards[Tiab] OR alligator[Tiab] OR alligators[Tiab] OR crocodile[Tiab] OR crocodiles[Tiab] OR turtle[Tiab] OR turtles[Tiab] OR amphibian[Tiab] OR amphibians[Tiab] OR amphibia[Tiab] OR frog[Tiab] OR frogs[Tiab] OR bombina[Tiab] OR salientia[Tiab] OR toad[Tiab] OR toads[Tiab] OR "epidalea calamita"[Tiab] OR salamander[Tiab] OR salamanders[Tiab] OR eel[Tiab] OR eels[Tiab] OR fish[Tiab] OR fishes[Tiab] OR pisces[Tiab] OR catfish[Tiab] OR catfishes[Tiab] OR siluriformes[Tiab] OR arius[Tiab] OR heteropneustes[Tiab] OR sheatfish[Tiab] OR perch[Tiab] OR perches[Tiab] OR percidae[Tiab] OR perca[Tiab] OR trout[Tiab] OR trouts[Tiab] OR char[Tiab] OR chars[Tiab] ORsalvelinus[Tiab] OR minnow[Tiab] OR cyprinidae[Tiab] OR carps[Tiab] OR carp[Tiab] OR zebrafish[Tiab] OR zebrafishes[Tiab] OR goldfish[Tiab] OR goldfishes[Tiab] OR guppy[Tiab] OR guppies[Tiab] OR chub[Tiab] OR chubs[Tiab] OR tinca[Tiab] OR barbels[Tiab] ORbarbus[Tiab] OR pimephales[Tiab] OR promelas[Tiab] OR "poecilia reticulata"[Tiab] OR mullet[Tiab] OR mullets[Tiab] OR eel[Tiab] OR eels[Tiab] OR seahorse[Tiab] OR seahorses[Tiab] OR mugil curema[Tiab] OR atlantic cod[Tiab] OR shark[Tiab] OR sharks[Tiab] OR catshark[Tiab] OR anguilla[Tiab] OR salmonid[Tiab] OR salmonids[Tiab] OR whitefish[Tiab] OR whitefishes[Tiab] OR salmon[Tiab] OR salmons[Tiab] OR sole[Tiab] OR solea[Tiab] OR lamprey[Tiab] OR lampreys[Tiab] OR pumpkinseed[Tiab] OR sunfish[Tiab] OR sunfishes[Tiab] OR tilapia[Tiab] OR tilapias[Tiab] OR turbot[Tiab] OR turbots[Tiab] OR flatfish[Tiab] OR flatfishes[Tiab] OR sciuridae[Tiab] OR squirrel[Tiab] OR squirrels[Tiab] OR chipmunk[Tiab] OR chipmunks[Tiab] OR suslik[Tiab] OR susliks[Tiab] OR vole[Tiab] OR voles[Tiab] OR lemming[Tiab] OR lemmings[Tiab] OR muskrat[Tiab] OR muskrats[Tiab] OR lemmus[Tiab] OR otter[Tiab] OR otters[Tiab] OR marten[Tiab] OR martens[Tiab] OR martes[Tiab] OR weasel[Tiab] OR badger[Tiab] OR badgers[Tiab] OR ermine[Tiab] OR mink[Tiab] OR minks[Tiab] OR sable[Tiab] OR sables[Tiab] OR gulo[Tiab] OR gulos[Tiab] OR wolverine[Tiab] OR wolverines[Tiab] OR mustela[Tiab] OR llama[Tiab] OR llamas[Tiab] OR alpaca[Tiab] OR alpacas[Tiab] OR camelid[Tiab] OR camelids[Tiab] OR guanaco[Tiab] OR guanacos[Tiab] OR chiroptera[Tiab] OR chiropteras[Tiab] OR bat[Tiab] OR bats[Tiab] OR fox[Tiab] OR foxes[Tiab] OR iguana[Tiab] OR iguanas[Tiab] OR xenopus laevis[Tiab] OR parakeet[Tiab] OR parakeets[Tiab] OR parrot[Tiab] OR parrots[Tiab] OR donkey[Tiab] OR donkeys[Tiab] OR mule[Tiab] OR mules[Tiab] OR zebra[Tiab] OR zebras[Tiab] OR shrew[Tiab] OR shrews[Tiab] OR bison[Tiab] OR bisons[Tiab] OR buffalo[Tiab] OR buffaloes[Tiab] OR deer[Tiab] OR deers[Tiab] OR bear[Tiab] OR bears[Tiab] OR panda[Tiab] OR pandas[Tiab] OR "wild hog"[Tiab] OR "wild boar"[Tiab] OR fitchew[Tiab] OR fitch[Tiab] OR beaver[Tiab] OR beavers[Tiab] OR jerboa[Tiab] OR jerboas[Tiab] OR capybara[Tiab] OR capybaras[Tiab]OR canine [tiab] OR bovine [tiab] OR porcine [tiab] OR hog [tiab] OR hogs [tiab]) NOT medline[sb]) |
| --- | --- |
| MRI | AND ("magnetic resonance imaging" OR "MRI") |
| Motor neuron disease | AND ("motor neuron disease" OR "amyotrophic lateral sclerosis" OR "ALS" OR "MND" OR "SOD") |

**Supplementary Table 2**: Summary of studies using magnetic resonance imaging (MRI) in motor neuron disease animal models (in alphabetical order).

*Glossary: ADC, apparent diffusion coefficient; ALS, amyotrophic lateral sclerosis;* DCE MRI, dynamic contrast-enhanced MRI; *DKI, diffusion kurtosis imaging; DTI, diffusion tensor imaging; DWI, diffusion-weighted imaging; FTD, frontotemporal dementia; MND, motor neuron disease; MTR, magnetization transfer ratio;* MRI, magnetic resonance imaging; *NODDI, neurite orientation dispersion and density imaging; T1w, T1-weighted; T2w, T2-weighted; T2*w, T2*-weighted; USPIO, ultra-small particles of iron oxide.*

| **Author, year** | **Title** | **Species** | **Model** | **MRI sequences** | **MRI field** | **nExp** | **nCtrl** | **Main findings** |
| --- | --- | --- | --- | --- | --- | --- | --- | --- |
| Andjus, 2009 | In vivo morphological changes in animal models of amyotrophic lateral sclerosis and Alzheimer's-like disease: MRI approach | Rat | G93A-SOD1 | T1w, T2w, Gd | 1.5T, 7T | Not reported | Not reported | T2w hyperintense foci in rat brain stem corresponded to astrogliosis. Rats showed dilation of lateral ventricles on MRI. Gd-DTPA application showed compromised blood-brain barrier adjacent to dilated lateral ventricles and in the perihippocampal region. |
| Angenstein, 2004 | Age-dependent changes in MRI of motor brain stem nuclei in a mouse model of ALS | Mouse | G93A-SOD1 (B6SJL) | T2w | 4.7T | 17 | 6 | MND model mice showed a time-dependent increase in signal intensities of nuclei V, VII, XII as well as nucleus ambiguus starting around day 90 which paralleled first behavioral signs of a motor neuron disorder. |
| Bataveljic, 2009 | Live monitoring of brain damage in the rat model of amyotrophic lateral sclerosis | Rat | G93A-SOD1 | T1w, T2w, T2*w, Gd | 1.5T | 15 | 0 | MND model rats showed T2w hyperintensities in the brainstem, rubrospinal tract as well as vagus motor nuclei with lateral ventricle and cerebral aqueduct enlargement. These were already observed in pre-symptomatic animals. |
| Bataveljic, 2011 | Imaging cellular markers of neuroinflammation in the brain of the rat model of amyotrophic lateral sclerosis | Rat | G93A-SOD1 | T1w, T2w, Gd | 1.5T | 9 | 0 | Study acquired MRI in an MND rat model upon application of USPIO labeled T-cells. This showed CD4+ lymphocyte infiltration in the midbrain whereas CD8+ cells were more confined to the brainstem. Gadolinium-enhanced studies exhibited blood-brain barrier leakage, congruent with T cell infiltration on USPIO MRI. Blood-brain barrier breakdown was congruent with the MRI foci of T-cell infiltration |
| Bigini, 2012 | Longitudinal tracking of human fetal cells labeled with super paramagnetic iron oxide nanoparticles in the brain of mice with motor neuron disease | Mouse | Wobbler | T2w | 7T | 24 | 24 | Study authors injected SPIOn-labelled human amniotic fluid cells into the ventricles of MND model mice or control mice. These cells spread to the whole ventricular system but did not migrate into the brain parenchyma. The cells did not alter survival of motor neuron disease mice. |
| Bonafede, 2020 | ASC-Exosomes Ameliorate the Disease Progression in SOD1(G93A) Murine Model Underlining Their Potential Therapeutic Use in Human ALS | Mouse | G93A-SOD1 (B6SJL) | T2*w | 4.7T | 8 | 8 | Study applied USPIO-labelled exosomes to motor neuron disease model mice demonstrating enhanced motor performance upon treatment. |
| Bontempi, 2018 | MRI reveals therapeutical efficacy of stem cells: An experimental study on the SOD1(G93A) animal model | Mouse | G93A-SOD1 (B6SJL) | T2w, T2 map, DTI | 4.7T | 26 | 14 | Motor neuron disease model mice show hyperintense gray matter changes in the brain stem, accompanied by increased T2 relaxation times. |
| Borg, 2008 | Differential MRI patterns of brain atrophy in double or single transgenic mice for APP and/or SOD | Mouse | SOD, APP or SOD/APP overexpressing mice | T2w | 7.05T | 84 | 0 | T2w MRI was employed to quantify the volume of brain regions of interest in transgenic mice overexpressing APP, SOD1, or both. In double transgenic mice, the most pronounced changes in gray matter volume were observed in 1-year-old double transgenic mice: Hippocampus, entorhinal as well as cingulate cortex volumes were decreased by 8% to 25%. In contrast, SOD1 transgenic mice showed atrophy specifically in cortical regions (cingulate, retrosplenial, and temporoparietal cortex), but no significant volume loss was found in the hippocampus. |
| Bucher, 2007 | Vacuolization correlates with spin-spin relaxation time in motor brainstem nuclei and behavioural tests in the transgenic G93A-SOD1 mouse model of ALS | Mouse | G93A-SOD1 (B6SJL) | T2w, T2 map | 4.7T | 12 | 7 | In the brainstem of motor neuron disease model mice, T2 values were increased in motor nuclei Nc. V, Nc. VII and Nc. XII as early as day 80, i.e., before the mean disease onset. On histopathology, this increase in signal was associated with a development of vacuoles in the brainstem motor nuclei and a decreased performance in behavioral tests. |
| Canzi, 2012 | Human skeletal muscle stem cell antiinflammatory activity ameliorates clinical outcome in amyotrophic lateral sclerosis models | Mouse | Wobbler | T1w, T2w, T2*w | 7T | 40 | 40 | In vivo tracking of USPIO-labelled human skeletal muscle-derived stem cells which were injected intracerebroventricularly. Transplanted cells were localized along the whole ventricular system which was also associated with enhanced motor performance of treated mice. |
| Caron, 2015 | Comparative Magnetic Resonance Imaging and Histopathological Correlates in Two SOD1 Transgenic Mouse Models of Amyotrophic Lateral Sclerosis | Mouse | G93A-SOD1 (B6.Cg) or 129Sv SOD1G93A | T2w, T2 map, DTI | 7T | 11 | 0 | A longitudinal in vivo analysis of T2 maps on cranial motor nuclei was performed. Increased T2 values were associated with tissue vacuolization that occurred prior to motor neuron loss in the cranial nuclei of C57 SOD1G93A mice. In contrast, 129Sv SOD1G93A mice, exhibiting a more severe phenotype, MRI detected a milder increase of T2 valuse, also associated with a milder vacuolization. |
| Choi, 2010 | Longitudinal monitoring of motor neuron circuitry in FALS rats using in-vivo phMRI | Rat | G93A-SOD1 | T2w/T2*w (pharmacologic MRI) | 9.4T | 7 | 0 | Using IRON (Increased Relaxivity for Optimized Neuroimaging), imaging was sensitized to cerebral blood volume changes. After baseline imaging, monocrystalline iron oxide nanoparticles (MION) were injected and subsequently amphetamine during continuous gradient echo imaging. With this, the study shows that pre-symptomatic motor neuron disease model rats had an activation pattern to amphetamine indistinguishable from wild-type controls. In contrast, symptomatic rats exhibited decreased response in sensorimotor cortex and increased response in M2 motor cortex, caudate/putamen, and thalamus. |
| Cowin, 2011 | Magnetic resonance microimaging of the spinal cord in the SOD1 mouse model of amyotrophic lateral sclerosis detects motor nerve root degeneration | Mouse | G93A-SOD1 | Magnetic resonance microscopy | 16.4T | 7 | 7 | Ex vivo ex situ study: in motor neuron disease model mice, magnetic resonance microscopy showed signal intensity increased by around 40% exclusively in the ventral motor nerve roots of the lumbar spinal cord compared to wildtype littermates. These hyperintensities were limited to white matter tracts arising from the motor neurons, whereas sensory white matter fibers were spared. Study also observed a decrease in ventral nerve root volume in the SOD1 mice, which correlated with the axonal degeneration observed by microscopy. |
| Evans, 2014 | CNS-targeted glucocorticoid reduces pathology in mouse model of amyotrophic lateral sclerosis | Mouse | G93A-SOD1 | T2w | 7T | 27 | 10 | Treatment with liposomal encapsulated glucocorticoid reduced T2w hyperintensities in brain stem nuclei of motor neuron disease model mice. |
| Evans, 2014 | T2-weighted MRI detects presymptomatic pathology in the SOD1 mouse model of ALS | Mouse | G93A-SOD1 | T1w, T2w, ADC, MTR | 7T | 9 | 9 | T2w MRI detected pre-symptomatic changes in brainstem nuclei in motor neuron disease model mice, as early as 60 days. Histologic correlation showed vacuolation, astro- and microglial activation correlating with T2w changes. Also, reductions in ADC and MTR were observed at 120 days in the same brainstem nuclei. No changes inT1 relaxation, vascular permeability, or endothelial activation were observed during any stage of the disease. |
| Figini, 2016 | Comparison of Diffusion MRI Acquisition Protocols for the In Vivo Characterization of the Mouse Spinal Cord: Variability Analysis and Application to an Amyotrophic Lateral Sclerosis Model | Mouse | G93A-SOD1 (B6SJL) | DWI | 7T | 7 | 7 | The purpose of this study was to compare three diffusion weighted acquisition protocols allowing a robust estimation of the main diffusion quantities to neurodegeneration in the mouse spinal cord. A diffusion weighted protocol with an intermediate number of diffusion gradient directions and a relatively high diffusion weighting was optimal for spinal cord imaging. |
| Gao, 2020 | Multicomponent diffusion analysis reveals microstructural alterations in spinal cord of a mouse model of amyotrophic lateral sclerosis ex vivo | Mouse | G93A-SOD1 (B6SJL) | DWI | 9.4T | 7 | 8 | Study aimed at investigating signal decay behaviors at ultra-high b-values for non-invasive assessment of spinal cord changes in the transgenic SOD1G93A mouse model. Anisotropic diffusion signals persisted at ultra-high b-value DWIs of the mouse spinal cord. Thus, multiexponential diffusion analysis offers the potential to evaluate microstructural alterations of ALS-affected spinal cord non-invasively |
| Gatto, 2019 | Unveiling early cortical and subcortical neuronal degeneration in ALS mice by ultra-high field diffusion MRI | Mouse | G93A-SOD1 (B6SJL) | DTI, DKI, NODDI | 16.7T | 15 | 3 | Study aimed at determining if Gaussian and non-Gaussian diffusion models gathered by ultra-high field diffusion MRI are appropriate to detect early structural changes in brain white and gray matter in a motor neuron disease model. The combination of DTI, NODDI, and DKI models have shown to provide a more complete assessment of the early microstructural changes in the ALS mouse brain, particularly in areas associated with high cognitive function |
| Gatto, 2018 | In vivo diffusion MRI detects early spinal cord axonal pathology in a mouse model of amyotrophic lateral sclerosis | Mouse | G93A-SOD1 (B6SJL) | DTI/DWI | 9.4T | 5 | 5 | Study investigated the sensitivity of DTI for the detection of signs of tissue damage before symptom onset. Changes were observed in fractional anisotropy, axial and radial diffusivity at postnatal day 80. These differences were associated with changes in axonal fluorescence intensity and membrane cellular markers |
| Gatto, 2018 | Diffusion tensor imaging identifies presymptomatic axonal degeneration in the spinal cord of ALS mice | Mouse | G93A-SOD1 (B6SJL) | DTI/DWI | 9.4T | 5 | 5 | Study investigated the sensitivity of DTI for the detection of signs of tissue damage before symptom onset. DTI studies showed a reduction in fractional anisotropy and axial diffusivities as well as an increase in radial diffusivity, mainly at lower segments of the spinal cord. Diffusion changes were associated with early and progressive alterations in axonal connectivity following a distal to proximal progression. Histopathological analysis tagging neuronal, axonal and glial cell markers showed presymptomatic alterations in spinal cord white and gray matter |
| Gatto, 2018 | Neurite orientation dispersion and density imaging can detect presymptomatic axonal degeneration in the spinal cord of ALS mice | Mouse | G93A-SOD1 (B6SJL) | NODDI | 17.6T | 40 | 0 | Ex vivo ex situ study. Study investigated the sensitivity of DTI for the detection of signs of tissue damage before symptom onset. NODDI showed a decrease in intra-cellular volume fraction as well as increases in orientation dispersion index/isotropic volume fraction. Furthermore, histopathology demonstrated a reduction in axonal area and myelin content |
| Grant, 2014 | Abnormalities in whisking behaviour are associated with lesions in brain stem nuclei in a mouse model of amyotrophic lateral sclerosis | Mouse | G93A-SOD1 (B6SJL) | T2w | 7T | 5 | 5 | Facial nucleus becoming more hyperintense in motor neuron disease model mice between 60 and 120 days |
| Grolez, 2018 | MRI of the cervical spinal cord predicts respiratory dysfunction in ALS | Mouse | G93A-SOD1 | T2w*2 | 7T | Not reported | Not reported | Motor neuron disease model mice showed increased R2* values in the cervical spinal cord as the first abnormality at day 85, just when the mice became symptomatic in the limbs. At day 100, a decrease in the volumes of the cervical spinal cord and the motor cortex was observed. Also, iron accumulation appeared in the cervical spinal cord at symptom onset but disappeared with disease progression |
| Jouroukhin, 2013 | NAP (davunetide) modifies disease progression in a mouse model of severe neurodegeneration: protection against impairments in axonal transport | Mouse | G93A-SOD1 | T1w, T2w, T2 map, manganese-enhanced MRI | 7T | 14 | 4 | Study aimed at evaluating the impact of davunetide (NAP, a neuroprotective agent) on axonal transport in a motor neuron disease animal model. Manganese-enhanced MRI (estimating axonal transport rates) showed a reduction of the anterograde axonal transport in the ALS mice whereas NAP treatment normalized axonal transport rates. Sequential MRI exhibited a damage to the ventral tegmental area (VTA). In contrast, daily NAP treatment delayed degeneration of the trigeminal, facial, and hypoglossal motor nuclei which was apparent at days 90–100 |
| Majchrzak, 2019 | SOD1/Rag2 Mice with Low Copy Number of SOD1 Gene as a New Long-Living Immunodeficient Model of ALS | Mouse | hSOD1/rag2 | T2w | 7T | Not reported | Not reported | MRI demonstrated T2w hyperintensities in the facial, parapyramidal as well as paragigantocellular reticular nuclei, mainly during the symptomatic period of the disease |
| Marcuzzo, 2017 | A longitudinal DTI and histological study of the spinal cord reveals early pathological alterations in G93A-SOD1 mouse model of amyotrophic lateral sclerosis | Mouse | G93A-SOD1 (B6SJL) | DTI | 7T | Not reported | Not reported | Study showed a gradient of degeneration in the spinal cord white and gray matter, starting in the ventral white matter. Study also found that even the main sensory regions are affected by the neurodegenerative process at symptomatic disease phase |
| Marcuzzo, 2011 | Hind limb muscle atrophy precedes cerebral neuronal degeneration in G93A-SOD1 mouse model of amyotrophic lateral sclerosis: a longitudinal MRI study | Mouse | G93A-SOD1 (B6SJL) | T2w | 7T | 7 | 7 | Study employed MRI to measure muscle atrophy and concomitant brain changes in a mouse motor neuron disease model. This longitudinal MRI study establishes that motor neuron disease model mice show muscular degeneration prior to evidence of neurodegeneration on MRI. Neurodegenerative changes on MRI were observed only in the motor nuclei areas of the brainstem, yet no changes indicative for neurodegeneration were detected in the motor cortex, even at the late disease stages |
| Moreau, 2018 | Could Conservative Iron Chelation Lead to Neuroprotection in Amyotrophic Lateral Sclerosis? | Mouse | 86R-SOD1 | T2*w | 7T | 30 | 10 | Study assessed safety and efficacy of conservative iron chelation in a murine motor neuron disease model. Iron levels, as measured by MRI (shown by a decrease in R2*), were lower upon deferiprone treatment in the cervical spinal cord, medulla oblongata and motor cortex |
| Niessen, 2006 | In vivo quantification of spinal and bulbar motor neuron degeneration in the G93A-SOD1 transgenic mouse model of ALS by T2 relaxation time and apparent diffusion coefficient | Mouse | G93A-SOD1 (B6SJL) | T2w, T2 map, ADC | 7T | 9 | 4 | Motor neuron disease model mice showed increased T2 values in the brain stem motor nuclei Nc. V (trigeminal nucleus), VII (facial nucleus), and XII (hypoglossal nucleus), and spinal cord. Similar effects in these motor nuclei were observed by ADC mapping. Additionally, in the upper spinal cord, a dorsal–ventral difference with higher T2 values in the ventral part was demonstrated by T2 mapping. |
| Underwood, 2011 | Non-invasive diffusion tensor imaging detects white matter degeneration in the spinal cord of a mouse model of amyotrophic lateral sclerosis | Mouse | G93A-SOD1 | DWI/DTI | 16.4T | 15 | 0 | Fractional anisotropy values were reduced exclusively in the ventral white matter tracts of the lumbar spinal cord of motor neuron disease model mice. This effect became stronger with increasing disease duration. |
| White, 2019 | Sarm1 deletion suppresses TDP-43-linked motor neuron degeneration and cortical spine loss | Mouse | Q331K-TDP43 | T2w | 9.4T | 32 | 0 | Study aimed at assessing whether the gene SARM1 could be a therapeutic target for ALS. With this, SARM1 was deleted from a murine ALS-FTD model. Ex vivo in situ MRI identified the entorhinal cortex as the region with most atrophy. Correlative histopathology confirmed a greater loss of neurons in the entorhinal cortex than in the motor cortex, indicating a prominent FTD-like pattern of neurodegeneration in this transgenic mouse model. SARM1 deletion did not mitigate age-related behavioral deficits. |
| Wilson, 2004 | Quantitative measurement of neurodegeneration in an ALS-PDC model using MR microscopy | Mouse | Mice fed with cycad toxins | T2*w (Magnetic resonance microscopy) | 17.6T | 7 | 7 | Motor neuron disease model mice showed decreased volumes in lumbar spinal cord gray matter, substantia nigra, striatum, basal nucleus/internal capsule, and olfactory bulb |
| Zang, 2004 | Magnetic resonance imaging reveals neuronal degeneration in the brainstem of the superoxide dismutase 1 transgenic mouse model of amyotrophic lateral sclerosis | Mouse | G93A-SOD1 (B6SJL) | T2w | 4.7T | 6 | 6 | Motor neuron disease model mice showed T2w hyperintensities in several regions including the nucleus ambiguus, facial nucleus, trigeminal motor nucleus, rostroventrolateral reticular nucleus, lateral paragigantocellular nucleus and the substantia nigra. Correlative histopathologytology showed vacuolar degeneration of respective structures. Furthermore, enlarged ventricles and hypointense striations, indicative of global atrophy, were also observed in the brain and cerebellum. |
| Zamani, 2022 | Impaired glymphatic function in the early stages of disease in a TDP-43 mouse model of amyotrophic lateral sclerosis | Mouse | hTDP-43ΔNLS | T2*w, DWI, DTI, DCE | 9.4T | 7 | 5 | Structural MRI showed progressive volume loess of the grey matter (the olfactory bulb, frontal association cortex, lateral and dorsolateral orbital cortex, agranular insular cortex, globus pallidus, hippocampus, dorsal subiculum, secondary visual cortex and cerebellum). DCE‑MRI showed altered dynamics of gadolinium clearance upon intracisternal injection. |

**Supplementary Table 3**: Risk of bias assessment of the included animal studies according to the risk of bias assessment checklist for good research practice[[3](#_ENREF_3)]: B, blinding; R, randomization; C, conflict of interest; S, prior sample size calculation; W, animal welfare statement.

| **Author, year** | **Title** | **B** | **R** | **C** | **S** | **W** |
| --- | --- | --- | --- | --- | --- | --- |
| Andjus, 2009 | In vivo morphological changes in animal models of amyotrophic lateral sclerosis and Alzheimer's-like disease: MRI approach | 0 | 0 | 0 | 0 | 1 |
| Angenstein, 2004 | Age-dependent changes in MRI of motor brain stem nuclei in a mouse model of ALS | 0 | 0 | 0 | 0 | 0 |
| Bataveljic, 2009 | Live monitoring of brain damage in the rat model of amyotrophic lateral sclerosis | 0 | 0 | 0 | 0 | 0 |
| Bataveljic, 2011 | Imaging cellular markers of neuroinflammation in the brain of the rat model of amyotrophic lateral sclerosis | 0 | 0 | 0 | 0 | 0 |
| Bigini, 2012 | Longitudinal tracking of human fetal cells labeled with super paramagnetic iron oxide nanoparticles in the brain of mice with motor neuron disease | 0 | 0 | 1 | 0 | 1 |
| Bonafede, 2020 | ASC-Exosomes Ameliorate the Disease Progression in SOD1(G93A) Murine Model Underlining Their Potential Therapeutic Use in Human ALS | 1 | 0 | 1 | 0 | 1 |
| Bontempi, 2018 | MRI reveals therapeutical efficacy of stem cells: An experimental study on the SOD1(G93A) animal model | 0 | 1 | 0 | 0 | 1 |
| Borg, 2008 | Differential MRI patterns of brain atrophy in double or single transgenic mice for APP and/or SOD | 0 | 0 | 0 | 0 | 1 |
| Bucher, 2007 | Vacuolization correlates with spin-spin relaxation time in motor brainstem nuclei and behavioural tests in the transgenic G93A-SOD1 mouse model of ALS | 0 | 0 | 0 | 0 | 1 |
| Canzi, 2012 | Human skeletal muscle stem cell antiinflammatory activity ameliorates clinical outcome in amyotrophic lateral sclerosis models | 0 | 0 | 1 | 0 | 1 |
| Caron, 2015 | Comparative Magnetic Resonance Imaging and Histopathological Correlates in Two SOD1 Transgenic Mouse Models of Amyotrophic Lateral Sclerosis | 1 | 1 | 1 | 0 | 1 |
| Choi, 2010 | Longitudinal monitoring of motor neuron circuitry in FALS rats using in-vivo phMRI | 0 | 0 | 0 | 0 | 1 |
| Cowin, 2011 | Magnetic resonance microimaging of the spinal cord in the SOD1 mouse model of amyotrophic lateral sclerosis detects motor nerve root degeneration | 0 | 0 | 0 | 0 | 1 |
| Evans, 2014 | CNS-targeted glucocorticoid reduces pathology in mouse model of amyotrophic lateral sclerosis | 1 | 1 | 1 | 0 | 1 |
| Evans, 2014 | T?-weighted MRI detects presymptomatic pathology in the SOD1 mouse model of ALS | 0 | 0 | 1 | 1 | 1 |
| Figini, 2016 | Comparison of Diffusion MRI Acquisition Protocols for the In Vivo Characterization of the Mouse Spinal Cord: Variability Analysis and Application to an Amyotrophic Lateral Sclerosis Model | 0 | 0 | 1 | 0 | 1 |
| Gao, 2020 | Multicomponent diffusion analysis reveals microstructural alterations in spinal cord of a mouse model of amyotrophic lateral sclerosis ex vivo | 0 | 0 | 1 | 0 | 1 |
| Gatto, 2019 | Unveiling early cortical and subcortical neuronal degeneration in ALS mice by ultra-high field diffusion MRI | 0 | 0 | 1 | 1 | 1 |
| Gatto, 2018 | In vivo diffusion MRI detects early spinal cord axonal pathology in a mouse model of amyotrophic lateral sclerosis | 0 | 0 | 1 | 0 | 1 |
| Gatto, 2018 | Diffusion tensor imaging identifies presymptomatic axonal degeneration in the spinal cord of ALS mice | 0 | 0 | 1 | 0 | 1 |
| Gatto, 2018 | Neurite orientation dispersion and density imaging can detect presymptomatic axonal degeneration in the spinal cord of ALS mice | 0 | 0 | 0 | 0 | 1 |
| Grant, 2014 | Abnormalities in whisking behaviour are associated with lesions in brain stem nuclei in a mouse model of amyotrophic lateral sclerosis | 0 | 0 | 0 | 1 | 1 |
| Grolez, 2018 | MRI of the cervical spinal cord predicts respiratory dysfunction in ALS | 1 | 0 | 1 | 0 | 1 |
| Jouroukhin, 2013 | NAP (davunetide) modifies disease progression in a mouse model of severe neurodegeneration: protection against impairments in axonal transport | 0 | 0 | 1 | 0 | 1 |
| Majchrzak, 2019 | SOD1/Rag2 Mice with Low Copy Number of SOD1 Gene as a New Long-Living Immunodeficient Model of ALS | 0 | 0 | 1 | 0 | 1 |
| Marcuzzo, 2017 | A longitudinal DTI and histological study of the spinal cord reveals early pathological alterations in G93A-SOD1 mouse model of amyotrophic lateral sclerosis | 0 | 0 | 1 | 0 | 1 |
| Marcuzzo, 2011 | Hind limb muscle atrophy precedes cerebral neuronal degeneration in G93A-SOD1 mouse model of amyotrophic lateral sclerosis: a longitudinal MRI study | 0 | 1 | 1 | 0 | 1 |
| Moreau, 2018 | Could Conservative Iron Chelation Lead to Neuroprotection in Amyotrophic Lateral Sclerosis? | 1 | 0 | 1 | 0 | 1 |
| Niessen, 2006 | In vivo quantification of spinal and bulbar motor neuron degeneration in the G93A-SOD1 transgenic mouse model of ALS by T2 relaxation time and apparent diffusion coefficient | 0 | 0 | 0 | 0 | 1 |
| Underwood, 2011 | Non-invasive diffusion tensor imaging detects white matter degeneration in the spinal cord of a mouse model of amyotrophic lateral sclerosis | 0 | 1 | 0 | 0 | 1 |
| White, 2019 | Sarm1 deletion suppresses TDP-43-linked motor neuron degeneration and cortical spine loss | 1 | 1 | 1 | 0 | 1 |
| Wilson, 2004 | Quantitative measurement of neurodegeneration in an ALS-PDC model using MR microscopy | 0 | 1 | 0 | 0 | 0 |
| Zang, 2004 | Magnetic resonance imaging reveals neuronal degeneration in the brainstem of the superoxide dismutase 1 transgenic mouse model of amyotrophic lateral sclerosis | 0 | 0 | 0 | 0 | 1 |
| Zamani, 2022 | Impaired glymphatic function in the early stages of disease in a TDP-43 mouse model of amyotrophic lateral sclerosis | 0 | 0 | 1 | 0 | 0 |

**Supplementary reference list**

In alphabetical order:

[1] P.R. Andjus, D. Bataveljić, G. Vanhoutte, D. Mitrecic, F. Pizzolante, N. Djogo, C. Nicaise, F. Gankam Kengne, C. Gangitano, F. Michetti, A. van der Linden, R. Pochet, and G. Bacić, In vivo morphological changes in animal models of amyotrophic lateral sclerosis and Alzheimer's-like disease: MRI approach. Anatomical record (Hoboken, N.J. : 2007) 292 (2009) 1882-92.

[2] F. Angenstein, H.G. Niessen, J. Goldschmidt, S. Vielhaber, A.C. Ludolph, and H. Scheich, Age-dependent changes in MRI of motor brain stem nuclei in a mouse model of ALS. Neuroreport 15 (2004) 2271-4.

[3] D. Bataveljić, N. Djogo, L. Zupunski, A. Bajić, C. Nicaise, R. Pochet, G. Bacić, and P.R. Andjus, Live monitoring of brain damage in the rat model of amyotrophic lateral sclerosis. General physiology and biophysics 28 (2009) 212-8.

[4] D. Bataveljić, S. Stamenković, G. Bačić, and P.R. Andjus, Imaging cellular markers of neuroinflammation in the brain of the rat model of amyotrophic lateral sclerosis. Acta physiologica Hungarica 98 (2011) 27-31.

[5] P. Bigini, V. Diana, S. Barbera, E. Fumagalli, E. Micotti, L. Sitia, A. Paladini, C. Bisighini, L. De Grada, L. Coloca, L. Colombo, P. Manca, P. Bossolasco, F. Malvestiti, F. Fiordaliso, G. Forloni, M. Morbidelli, M. Salmona, D. Giardino, T. Mennini, D. Moscatelli, V. Silani, and L. Cova, Longitudinal tracking of human fetal cells labeled with super paramagnetic iron oxide nanoparticles in the brain of mice with motor neuron disease. PloS one 7 (2012) e32326.

[6] R. Bonafede, E. Turano, I. Scambi, A. Busato, P. Bontempi, F. Virla, L. Schiaffino, P. Marzola, B. Bonetti, and R. Mariotti, ASC-Exosomes Ameliorate the Disease Progression in SOD1(G93A) Murine Model Underlining Their Potential Therapeutic Use in Human ALS. International journal of molecular sciences 21 (2020).

[7] P. Bontempi, A. Busato, R. Bonafede, L. Schiaffino, I. Scambi, A. Sbarbati, R. Mariotti, and P. Marzola, MRI reveals therapeutical efficacy of stem cells: An experimental study on the SOD1(G93A) animal model. Magnetic resonance in medicine 79 (2018) 459-469.

[8] J. Borg, and E. Chereul, Differential MRI patterns of brain atrophy in double or single transgenic mice for APP and/or SOD. Journal of neuroscience research 86 (2008) 3275-84.

[9] S. Bucher, K.E. Braunstein, H.G. Niessen, T. Kaulisch, M. Neumaier, T.M. Boeckers, D. Stiller, and A.C. Ludolph, Vacuolization correlates with spin-spin relaxation time in motor brainstem nuclei and behavioural tests in the transgenic G93A-SOD1 mouse model of ALS. The European journal of neuroscience 26 (2007) 1895-901.

[10] L. Canzi, V. Castellaneta, S. Navone, S. Nava, M. Dossena, I. Zucca, T. Mennini, P. Bigini, and E.A. Parati, Human skeletal muscle stem cell antiinflammatory activity ameliorates clinical outcome in amyotrophic lateral sclerosis models. Molecular medicine (Cambridge, Mass.) 18 (2012) 401-11.

[11] I. Caron, E. Micotti, A. Paladini, G. Merlino, L. Plebani, G. Forloni, M. Modo, and C. Bendotti, Comparative Magnetic Resonance Imaging and Histopathological Correlates in Two SOD1 Transgenic Mouse Models of Amyotrophic Lateral Sclerosis. PloS one 10 (2015) e0132159.

[12] J.K. Choi, A. Dedeoglu, and B.G. Jenkins, Longitudinal monitoring of motor neuron circuitry in FALS rats using in-vivo phMRI. Neuroreport 21 (2010) 157-62.

[13] G.J. Cowin, T.J. Butler, N.D. Kurniawan, C. Watson, and R.H. Wallace, Magnetic resonance microimaging of the spinal cord in the SOD1 mouse model of amyotrophic lateral sclerosis detects motor nerve root degeneration. NeuroImage 58 (2011) 69-74.

[14] M.C. Evans, P.J. Gaillard, M. de Boer, C. Appeldoorn, R. Dorland, N.R. Sibson, M.R. Turner, D.C. Anthony, and H.B. Stolp, CNS-targeted glucocorticoid reduces pathology in mouse model of amyotrophic lateral sclerosis. Acta neuropathologica communications 2 (2014) 66.

[15] M.C. Evans, S. Serres, A.A. Khrapitchev, H.B. Stolp, D.C. Anthony, K. Talbot, M.R. Turner, and N.R. Sibson, T₂-weighted MRI detects presymptomatic pathology in the SOD1 mouse model of ALS. Journal of cerebral blood flow and metabolism : official journal of the International Society of Cerebral Blood Flow and Metabolism 34 (2014) 785-93.

[16] M. Figini, A. Scotti, S. Marcuzzo, S. Bonanno, F. Padelli, V. Moreno-Manzano, J.M. García-Verdugo, P. Bernasconi, R. Mantegazza, M.G. Bruzzone, and I. Zucca, Comparison of Diffusion MRI Acquisition Protocols for the In Vivo Characterization of the Mouse Spinal Cord: Variability Analysis and Application to an Amyotrophic Lateral Sclerosis Model. PloS one 11 (2016) e0161646.

[17] J. Gao, M. Jiang, R.L. Magin, R.G. Gatto, G. Morfini, A.C. Larson, and W. Li, Multicomponent diffusion analysis reveals microstructural alterations in spinal cord of a mouse model of amyotrophic lateral sclerosis ex vivo. PloS one 15 (2020) e0231598.

[18] R.G. Gatto, M. Amin, A. Finkielsztein, C. Weissmann, T. Barrett, C. Lamoutte, O. Uchitel, R. Sumagin, T.H. Mareci, and R.L. Magin, Unveiling early cortical and subcortical neuronal degeneration in ALS mice by ultra-high field diffusion MRI. Amyotrophic lateral sclerosis & frontotemporal degeneration 20 (2019) 549-561.

[19] R.G. Gatto, W. Li, J. Gao, and R.L. Magin, In vivo diffusion MRI detects early spinal cord axonal pathology in a mouse model of amyotrophic lateral sclerosis. NMR in biomedicine 31 (2018) e3954.

[20] R.G. Gatto, W. Li, and R.L. Magin, Diffusion tensor imaging identifies presymptomatic axonal degeneration in the spinal cord of ALS mice. Brain research 1679 (2018) 45-52.

[21] R.G. Gatto, S.M. Mustafi, M.Y. Amin, T.H. Mareci, Y.C. Wu, and R.L. Magin, Neurite orientation dispersion and density imaging can detect presymptomatic axonal degeneration in the spinal cord of ALS mice. Functional neurology 33 (2018) 155-163.

[22] R.A. Grant, P.S. Sharp, A.J. Kennerley, J. Berwick, A. Grierson, T. Ramesh, and T.J. Prescott, Abnormalities in whisking behaviour are associated with lesions in brain stem nuclei in a mouse model of amyotrophic lateral sclerosis. Behavioural brain research 259 (2014) 274-83.

[23] G. Grolez, M. Kyheng, R. Lopes, C. Moreau, K. Timmerman, F. Auger, G. Kuchcinski, A. Duhamel, P. Jissendi-Tchofo, P. Besson, C. Laloux, M. Petrault, J.C. Devedjian, T. Pérez, P.F. Pradat, L. Defebvre, R. Bordet, V. Danel-Brunaud, and D. Devos, MRI of the cervical spinal cord predicts respiratory dysfunction in ALS. Scientific reports 8 (2018) 1828.

[24] Y. Jouroukhin, R. Ostritsky, Y. Assaf, G. Pelled, E. Giladi, and I. Gozes, NAP (davunetide) modifies disease progression in a mouse model of severe neurodegeneration: protection against impairments in axonal transport. Neurobiology of disease 56 (2013) 79-94.

[25] M. Majchrzak, K. Drela, A. Andrzejewska, P. Rogujski, S. Figurska, M. Fiedorowicz, P. Walczak, M. Janowski, B. Lukomska, and L. Stanaszek, SOD1/Rag2 Mice with Low Copy Number of SOD1 Gene as a New Long-Living Immunodeficient Model of ALS. Scientific reports 9 (2019) 799.

[26] S. Marcuzzo, S. Bonanno, M. Figini, A. Scotti, I. Zucca, L. Minati, N. Riva, T. Domi, A. Fossaghi, A. Quattrini, B. Galbardi, S. D'Alessandro, M.G. Bruzzone, J.M. García-Verdugo, V. Moreno-Manzano, R. Mantegazza, and P. Bernasconi, A longitudinal DTI and histological study of the spinal cord reveals early pathological alterations in G93A-SOD1 mouse model of amyotrophic lateral sclerosis. Experimental neurology 293 (2017) 43-52.

[27] S. Marcuzzo, I. Zucca, A. Mastropietro, N.K. de Rosbo, P. Cavalcante, S. Tartari, S. Bonanno, L. Preite, R. Mantegazza, and P. Bernasconi, Hind limb muscle atrophy precedes cerebral neuronal degeneration in G93A-SOD1 mouse model of amyotrophic lateral sclerosis: a longitudinal MRI study. Experimental neurology 231 (2011) 30-7.

[28] C. Moreau, V. Danel, J.C. Devedjian, G. Grolez, K. Timmerman, C. Laloux, M. Petrault, F. Gouel, A. Jonneaux, M. Dutheil, C. Lachaud, R. Lopes, G. Kuchcinski, F. Auger, M. Kyheng, A. Duhamel, T. Pérez, P.F. Pradat, H. Blasco, C. Veyrat-Durebex, P. Corcia, P. Oeckl, M. Otto, L. Dupuis, G. Garçon, L. Defebvre, Z.I. Cabantchik, J. Duce, R. Bordet, and D. Devos, Could Conservative Iron Chelation Lead to Neuroprotection in Amyotrophic Lateral Sclerosis? Antioxidants & redox signaling 29 (2018) 742-748.

[29] H.G. Niessen, F. Angenstein, K. Sander, W.S. Kunz, M. Teuchert, A.C. Ludolph, H.J. Heinze, H. Scheich, and S. Vielhaber, In vivo quantification of spinal and bulbar motor neuron degeneration in the G93A-SOD1 transgenic mouse model of ALS by T2 relaxation time and apparent diffusion coefficient. Experimental neurology 201 (2006) 293-300.

[30] C.K. Underwood, N.D. Kurniawan, T.J. Butler, G.J. Cowin, and R.H. Wallace, Non-invasive diffusion tensor imaging detects white matter degeneration in the spinal cord of a mouse model of amyotrophic lateral sclerosis. NeuroImage 55 (2011) 455-61.

[31] M.A. White, Z. Lin, E. Kim, C.M. Henstridge, E. Pena Altamira, C.K. Hunt, E. Burchill, I. Callaghan, A. Loreto, H. Brown-Wright, R. Mead, C. Simmons, D. Cash, M.P. Coleman, and J. Sreedharan, Sarm1 deletion suppresses TDP-43-linked motor neuron degeneration and cortical spine loss. Acta neuropathologica communications 7 (2019) 166.

[32] J.M. Wilson, M.S. Petrik, S.C. Grant, S.J. Blackband, J. Lai, and C.A. Shaw, Quantitative measurement of neurodegeneration in an ALS-PDC model using MR microscopy. NeuroImage 23 (2004) 336-43.

[33] A. Zamani, A.K. Walker, B. Rollo, K.L. Ayers, R. Farah, T.J. O'Brien, and D.K. Wright, Impaired glymphatic function in the early stages of disease in a TDP-43 mouse model of amyotrophic lateral sclerosis. Transl Neurodegener 11 (2022) 17.

[34] D.W. Zang, Q. Yang, H.X. Wang, G. Egan, E.C. Lopes, and S.S. Cheema, Magnetic resonance imaging reveals neuronal degeneration in the brainstem of the superoxide dismutase 1 transgenic mouse model of amyotrophic lateral sclerosis. The European journal of neuroscience 20 (2004) 1745-51.

**References**

[1] C.R. Hooijmans, A. Tillema, M. Leenaars, and M. Ritskes-Hoitinga, Enhancing search efficiency by means of a search filter for finding all studies on animal experimentation in PubMed. Laboratory animals 44 (2010) 170-5.

[2] R.B. de Vries, C.R. Hooijmans, A. Tillema, M. Leenaars, and M. Ritskes-Hoitinga, Updated version of the Embase search filter for animal studies. Laboratory animals 48 (2014) 88.

[3] M.R. Macleod, M. Fisher, V. O'Collins, E.S. Sena, U. Dirnagl, P.M. Bath, A. Buchan, H.B. van der Worp, R.J. Traystman, K. Minematsu, G.A. Donnan, and D.W. Howells, Reprint: Good laboratory practice: preventing introduction of bias at the bench. Journal of cerebral blood flow and metabolism : official journal of the International Society of Cerebral Blood Flow and Metabolism 29 (2009) 221-3.
